# Cancer-associated fibroblasts: protagonists of the tumor microenvironment in gastric cancer

**DOI:** 10.3389/fmolb.2024.1340124

**Published:** 2024-03-18

**Authors:** Ece Ozmen, Tevriz Dilan Demir, Gulnihal Ozcan

**Affiliations:** ^1^ Koç University Graduate School of Health Sciences, Istanbul, Türkiye; ^2^ Koç University Research Center for Translational Medicine (KUTTAM), Istanbul, Türkiye; ^3^ Department of Medical Pharmacology, Koç University School of Medicine, Istanbul, Türkiye

**Keywords:** cancer-associated fibroblasts, tumor microenvironment, gastric cancer, chemoresistance, stemness, metastasis

## Abstract

Enhanced knowledge of the interaction of cancer cells with their environment elucidated the critical role of tumor microenvironment in tumor progression and chemoresistance. Cancer-associated fibroblasts act as the protagonists of the tumor microenvironment, fostering the metastasis, stemness, and chemoresistance of cancer cells and attenuating the anti-cancer immune responses. Gastric cancer is one of the most aggressive cancers in the clinic, refractory to anti-cancer therapies. Growing evidence indicates that cancer-associated fibroblasts are the most prominent risk factors for a poor tumor immune microenvironment and dismal prognosis in gastric cancer. Therefore, targeting cancer-associated fibroblasts may be central to surpassing resistance to conventional chemotherapeutics, molecular-targeted agents, and immunotherapies, improving survival in gastric cancer. However, the heterogeneity in cancer-associated fibroblasts may complicate the development of cancer-associated fibroblast targeting approaches. Although single-cell sequencing studies started dissecting the heterogeneity of cancer-associated fibroblasts, the research community should still answer these questions: “What makes a cancer-associated fibroblast protumorigenic?”; “How do the intracellular signaling and the secretome of different cancer-associated fibroblast subpopulations differ from each other?”; and “Which cancer-associated fibroblast subtypes predominate specific cancer types?”. Unveiling these questions can pave the way for discovering efficient cancer-associated fibroblast targeting strategies. Here, we review current knowledge and perspectives on these questions, focusing on how CAFs induce aggressiveness and therapy resistance in gastric cancer. We also review potential therapeutic approaches to prevent the development and activation of cancer-associated fibroblasts via inhibition of CAF inducers and CAF markers in cancer.

## 1 Introduction

Cancer is a highly dynamic and complex disease that evolves through the dynamic interaction of cancer cells with the tumor microenvironment (TME). The TME acts as a fertile soil to support the proliferation of cancer cells (de Visser and Joyce, 2023). To further feed this fertile soil, cancer cells secrete soluble factors like chemokines, cytokines, and growth factors to actively recruit diverse cell types to the TME and reprogram them (Mao et al., 2021). In turn, these cells in the TME facilitate the invasion and migration of cancer cells, their evasion from immune destruction, resistance to chemotherapy, and stimulation of angiogenesis, augmenting the tumor aggressiveness (de Visser and Joyce, 2023; Zhu et al., 2023). Hence, a complete understanding of how cancer cells interact with their microenvironment is crucial to overcoming current challenges in cancer treatment.

The TME comprises a diverse array of cellular and non-cellular components, interacting dynamically. The cellular components involve immune cells, vascular endothelial cells, pericytes, mesenchymal stem cells, adipocytes, and cancer-associated fibroblasts (CAFs) (de Visser and Joyce, 2023). Each type of these cells interacts with the tumor cells via cell-to-cell contact and paracrine signaling. Hence, a supportive environment for tumor growth, progression, metastasis, and chemoresistance is established. The non-cellular components of the TME are primarily comprised of the extracellular matrix (ECM). Embedding the cellular elements, intercellular signaling molecules (i.e., cytokines, growth factors, and chemokines), extracellular vesicles, and metabolites, ECM constitutes a dynamic and supportive framework for cancer cells (Sükei et al., 2021).

CAFs are the vital cellular elements in TME, orchestrating the dynamic and adaptable nature of the TME and playing a pivotal role in promoting tumor growth (Zhang et al., 2023a). CAFs are specialized fibroblasts found within the TME. They organize and modify both the cellular and the noncellular components of TME to reinforce the stromal barrier against the efficacy of anti-cancer therapies and immune cells. CAF infiltration is associated with poor prognosis in several cancers. Therefore, the interest in exploring the mechanisms by which CAFs induce a pro-tumorigenic TME is ever-growing in cancer research (Belhabib et al., 2021).

Gastric cancer is one of the cancers in which CAFs predominate the TME and lead to an aggressive phenotype. Zeng et al. investigated the TME infiltration pattern in a comprehensive cohort of gastric tumor samples. They demonstrated that CAF infiltration is the primary risk factor associated with a poor TME immune phenotype and dismal prognosis in gastric cancer patients (Zeng et al., 2019). Our analysis of comprehensive patient cohorts also illustrated that gastric cancer is one of the cancers in which CAF markers emerge as the primary biomarkers and are associated with the poorest prognosis (Ucaryilmaz Metin and Ozcan, 2022a). Therefore, it is crucial to delineate the dynamic interplay of CAFs with all the TME components and cancer cells to discover CAF-targeting therapies and improve survival in gastric cancer. Hence, here we review the role of CAFs in organizing the TME and inducing the progression and resistance of cancer cells to therapy with a focus on gastric cancer.

## 2 The origins of cancer-associated fibroblasts in the tumor microenvironment

**FIGURE 1:**
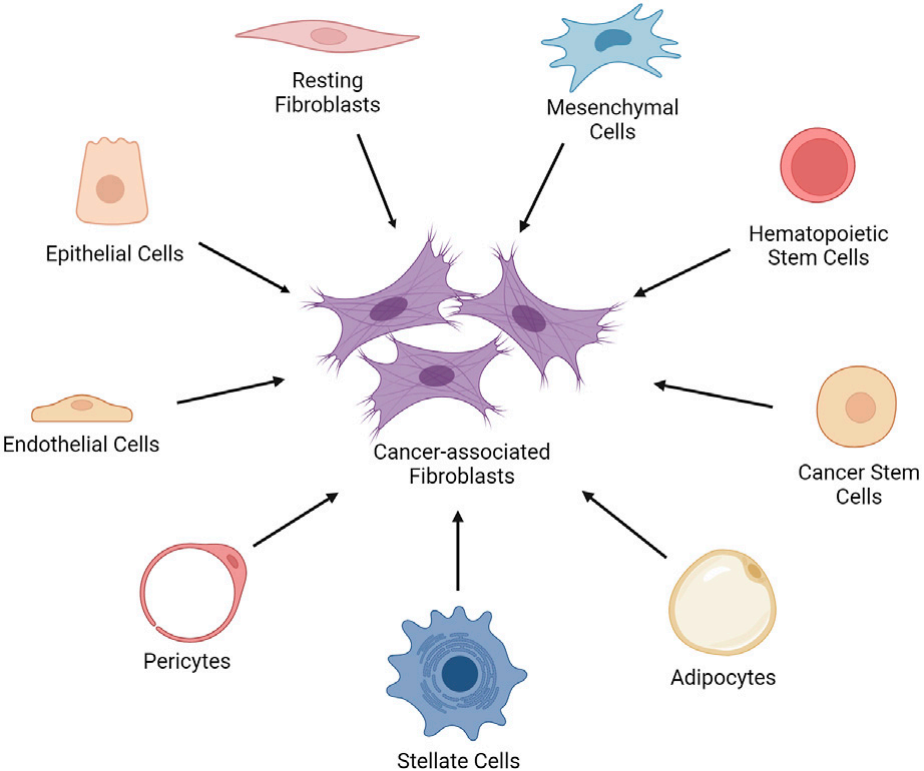
The cell types that give rise to cancer-associated fibroblasts. The cancer-associated fibroblasts (CAFs) can be generated by the epithelial-mesenchymal transition of activated fibroblasts, endothelial cells, and epithelial cells in the TME. Moreover, bone marrow-derived mesenchymal stem cells (MSCs), hematopoietic stem cells (HSCs) and cancer stem cells (CSCs), adipocytes, pericytes, and stellate cells can give rise to CAFs. Created with BioRender.com.

CAFs originate from diverse cell types in the TME (Figure 1). They can be generated by the epithelial-mesenchymal transition (EMT) of activated fibroblasts, endothelial cells, and epithelial cells. Additionally, bone marrow-derived mesenchymal stem cells (MSCs), hematopoietic stem cells (HSCs) and cancer stem cells (CSCs), adipocytes, pericytes, and stellate cells can give rise to CAFs (Xing et al., 2010).

EMT plays a key role not only in tumor progression but also in the formation of CAFs. Transforming growth factor-beta (TGF-β) and platelet-derived growth factor (PDGF) secreted by various TME cells act as the main factors in promoting the transition of resting epithelial cells and mesenchymal stem cells into CAFs (Quante et al., 2011; Baulida, 2017; Aoto et al., 2018). In dysplastic gastritis, increased expression of TGF-β by neighboring stromal cells was shown to induce the trans-differentiation of primarily local epithelial cells into myofibroblasts, which are CAF subtypes with contractile properties involved in tissue remodeling and ECM production (Fuyuhiro et al., 2011).

Genetic alterations are another common mechanism for the formation of CAFs from fibroblasts and epithelial cells (Louault et al., 2020). Gain of function mutations in oncogenes RAS and Myc or loss of function mutations in tumor suppressor genes p53 and PTEN in epithelial cells within the TME lead to the acquisition of CAF-like characteristics (Xing et al., 2010; Janssen et al., 2022). The inactivation of p53 was also shown to act as a positive regulator of the transition of resting fibroblasts into CAFs (Arandkar et al., 2018).

A closer examination of the origin of CAFs demonstrated that mesenchymal cells can also be the precursor of CAFs (Xing et al., 2010). Mesenchymal cells *in vitro* can transdifferentiate into CAFs when exposed to the conditioned media from tumor cells. The *in vivo* evidence of this trans-differentiation was also shown in mouse models. In an inflammation-induced gastric cancer mouse model, around 20% of CAFs originated from mesenchymal cells. Mesenchymal-derived bone marrow cells were observed to transform towards a CAF phenotype under the influence of TGF-β and PDGF (Quante et al., 2011).

Endothelial cells give rise to around 40% of the CAFs in tumors (Nie et al., 2014). Primarily, TGF-β and SMAD signaling pathways are involved in the transition of endothelial cells to CAFs. The binding of TGF-β to its receptors on endothelial cells triggers downstream SMAD-dependent signaling that ultimately leads to the transformation of these cells into a CAF-like phenotype. The intricate interplay between endothelial cells and CAFs underscores the dynamic nature of the tumor microenvironment and highlights the multifaceted contributions of different cell types to tumorigenesis and disease progression (van Meeteren and ten Dijke, 2012; Huang et al., 2023a).

## 3 Crosstalk between cancer cells and cancer-associated fibroblasts in the tumor microenvironment

The intricate bidirectional communication between cancer cells and CAFs within the TME constitutes a dynamic interplay crucial for understanding tumor development. In this complex interaction, both cell types influence each other, orchestrating a series of events that significantly impact cancer progression. Notably, CAFs are pivotal in supporting cancer cell metastasis, invasion, and overall tumor progression within the TME. Conversely, the formation and activation of CAFs are reciprocally induced by the signals emanating from cancer cells. In other words, cancer cells activate the assistance of CAFs to provide a conducive microenvironment for tumor development (Ping et al., 2021).

To investigate the impact of paracrine cross-talk of cancer-associated fibroblasts (CAFs) and cancer cells, Fozatti et al. exposed both cell types to conditioned media obtained sequentially from the other (Fozzatti et al., 2019). They observed that signaling molecules released by tumor cells, such as reactive oxygen species (ROS), PDGF, and IL-6, have the potential to alter the metabolism, characteristics, and secretory profile of fibroblasts, causing them to transit to CAF phenotypes (Fozzatti and Cheng, 2020). Concurrently, these activated fibroblasts released soluble factors that influence the phenotype of tumor epithelial cells, promoting cell proliferation and invasion in thyroid cancer cells, thereby enhancing the progression of thyroid cancer. Their findings imply the existence of a paracrine loop between tumor cells and stromal fibroblasts in thyroid cancer, ultimately increasing the aggressiveness of thyroid cancer (Fozzatti et al., 2019).

In addition to paracrine signaling, extracellular vesicles and exosomes secreted from cancer cells are crucial in CAF activation and recruitment in tumor sites. Vu et al. demonstrated that extracellular vesicles released by tumors play a pivotal role in activating CAFs through the transmission of microRNA-125b, a molecule strongly linked to poor prognosis in triple-negative breast cancer (Vu et al., 2019). To investigate this mechanism, the researchers fluorescently labeled and harvested extracellular vesicles from the highly metastatic 4T1 mouse breast cancer cell lines. These labeled vesicles were then introduced into the conditional media of poorly metastatic 4TO7 cells. They revealed that the extracellular vesicles released by tumors significantly contribute to the activation of CAFs by facilitating the transfer of microRNA-125b. Afterward, Yang et al. aimed to demonstrate the regulatory impact of cancer-derived exosomes on cell invasion and metastasis in breast cancer (Yang et al., 2020). Coculture models involving MCF-7 breast cancer cells and cancer cell-derived exosomes indicated that these exosomes promote the transformation of normal fibroblasts (NFs) into CAFs and enhance the recruitment of CAFs to MCF-7 cells.

Cancer cells stimulate the generation of CAFs, and the interplay between cancer cells and the TME also affects the plasticity and phenotype of the already-developed CAFs (Yang et al., 2023). To investigate the influence of cancer cells with high metastatic potential on the metabolic reprogramming of fibroblasts, Kogure et al. utilized transcriptome data from diffuse-type gastric cancer cells (Kogure et al., 2020). They found that glycolysis-related genes, including lactate dehydrogenase A (LDHA) and enolase 2 (ENO2) expression profiles significantly changed between fibroblasts cocultured with highly metastatic cancer cells and low metastatic potential cancer cells. The coculture with highly metastatic gastric cancer cells induced changes in glucose uptake, lactate production, and oxygen consumption in fibroblasts and favored aerobic glycolysis in stomach fibroblasts (Kogure et al., 2020). Aerobic glycolysis, also known as the Warburg effect, is a metabolic phenomenon characterized by the increased utilization of glycolysis even in the presence of oxygen (Liberti and Locasale, 2016). CAFs undergoing aerobic glycolysis play a supportive role in tumor growth (Kogure et al., 2020).

Several studies report the crosstalk between cancer cells and CAFs also in gastric cancer. Hong et al. reported that TGF-β1 derived from gastric cancer cells activated the *P*-Smad2/3 pathway in normal fibroblasts leading to their transformation into CAFs with a high expression of fibroblast activation protein (FAP), platelet-derived growth factor receptor beta (PDGFRB), and alpha-smooth muscle actin (α-SMA). Then, increased secretion of insulin-like growth factor binding protein-7 (IGFBP7) from these CAFs augmented gastric cancer cells’ migration and invasion capabilities and induced stemness. Transcriptome data of gastric cancer patients showed that high expression of IGFBP7 was correlated with a high infiltrating pattern and poorly differentiated phenotype, which exhibit poor prognosis (Hong et al., 2023). In another study, IL-1α, IL-1β, and tumor necrosis factor (TNF) secreted from diffuse gastric cancer cells were shown to increase the Rhomboid 5 homolog 2 (RHBDF2) in gastric fibroblasts, which induced a CAF phenotype in these fibroblasts increasing their motility. The authors demonstrated that RHBDF2 regulates TGF-β signaling, and CAFs with high RHBDF2 expression induce lymphatic invasion of gastric cancer cells in a mouse model (Ishimoto et al., 2017). Conversion of latent TGF-β released from normal fibroblasts into active TGF-β via urokinase-type plasminogen activator secreted by gastric cancer cells was also suggested as a mechanism by which gastric cancer cells induce differentiation of fibroblast into CAFs (Miki et al., 2020). Moreover, Zhu et al. reported a crosstalk between gastric cancer-derived mesenchymal stem cells (GC-MSCs) and neutrophils, which culminates in the transformation of MSCs to CAFs. The authors showed that GC-MSCs activated neutrophils through secreting IL-6 and activating the STAT3 and ERK pathways. In turn, activated neutrophils transformed MSCs into CAFs (Zhu et al., 2014). These findings indicate an intricate and dynamic bidirectional communication between cancer cells and CAFs within the TME. Understanding and unraveling the complexities of this multifaceted interplay within the TME can shed light on potential therapeutic targets and avenues for disrupting the supportive microenvironment in gastric cancer.

## 4 Functions of the cancer-associated fibroblasts in the tumor microenvironment

CAFs exhibit several distinctive features, distinguishing them from normal fibroblasts within the TME. They are more active than normal fibroblasts, with an enhanced proliferative and migratory capacity (Glabman et al., 2022). Morphologically, they are generally larger with indented nuclei and branched cytoplasm, lacking lineage markers of originating cells. The secretome of CAFs also differs from the originating cells. FAP, α-SMA, vimentin, and platelet-derived growth factor receptor alpha (PDGFR-α) are the primary markers that identify and characterize CAFs (Xing et al., 2010; Mao et al., 2021).

The altered secretory profiles of CAFs contribute to their pro-tumorigenic characteristics and influence on nearby cells. Secretion of various growth factors and cytokines such as TGF-β, fibroblast growth factor (FGF), PDGF, IL-6, and IL-8 by CAFs promotes cancer cell proliferation (Mao et al., 2021; Ping et al., 2021). Remarkably, when CAFs secrete TGF-β, they bind to its specific receptor on the surface of cancer cells and activate SMAD proteins (Wu et al., 2021). SMAD complex acts as a transcription factor in cancer cells and regulates the expression of genes like Cyclin D1, BCL-2, and Vascular Endothelial Growth Factor (VEGF) (Yang et al., 2021).

**FIGURE 2:**
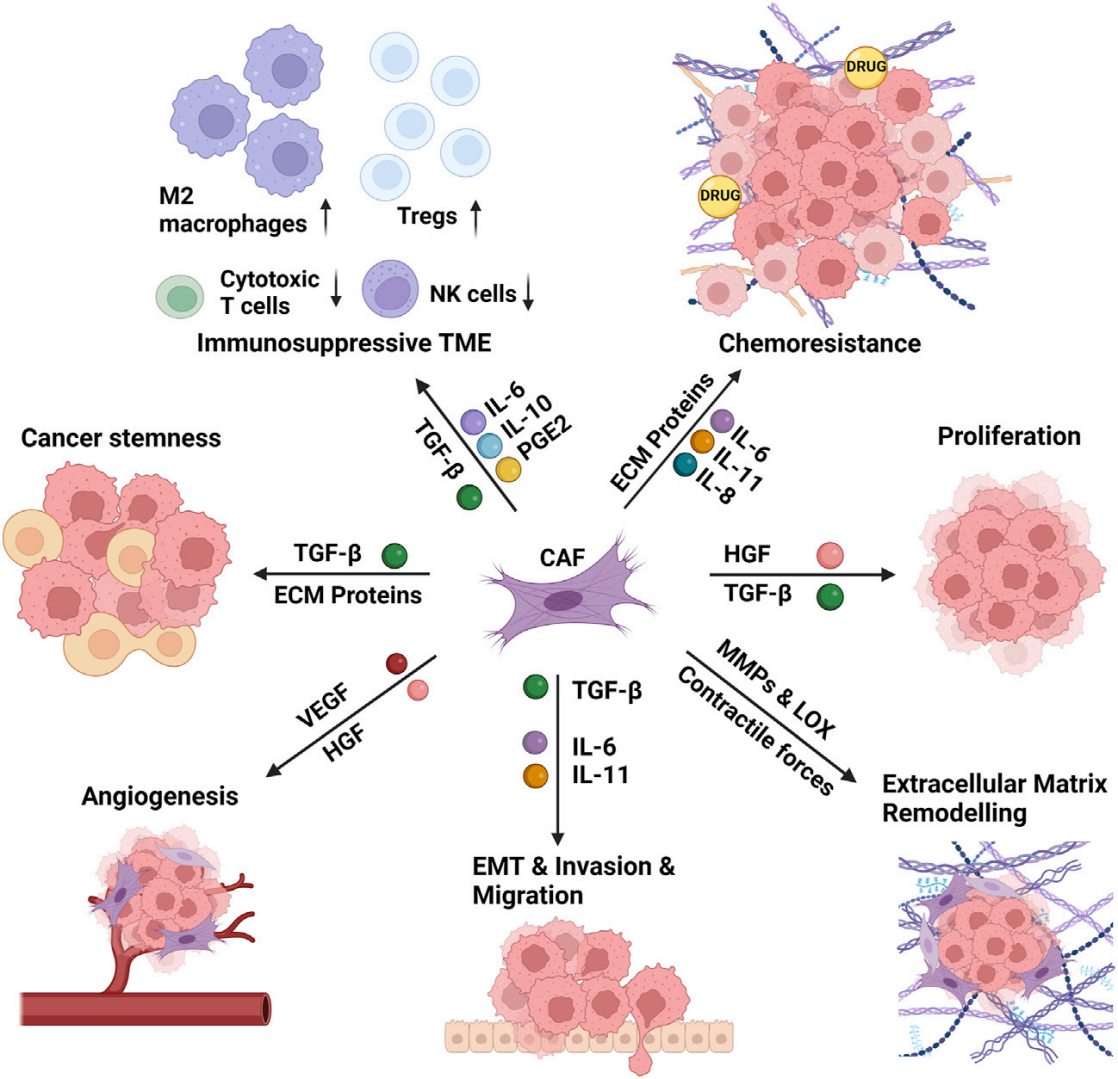
The functions of the cancer-associated fibroblasts in the tumor microenvironment. The cancer-associated fibroblasts (CAFs) induce the proliferation and stemness of cancer cells, remodel the extracellular matrix (ECM), and promote epithelial-mesenchymal transition (EMT) and invasion of surrounding tissues. CAF-induced matrix stiffness leads to chemoresistance via preventing the diffusion of anti-cancer drugs to the tumor tissue. CAFs also lead to an immunosuppressive microenvironment via increasing the recruitment and activity of M2 macrophages and regulatory T cells (Tregs) and decreasing the recruitment and activity of cytotoxic T cells and natural killer (NK) cells. CAFs perform these functions via secretion of several interleukins (IL-6, IL-8, IL-10, IL-11), growth factors (TGF-β: transforming growth factor- β, HGF: hepatocyte growth factor, VEGF: vascular endothelial growth factor), ECM proteins and ECM remodeling enzymes (MMPs: matrix metalloproteinases, LOX: lysyl oxidase), and several other mediators (e.g., PGE2: prostaglandin E2) and chemokines. Created with BioRender.com.

Secretion of a vast array of mediators from CAFs, including interleukins, growth factors, and chemokines, induce the proliferation and stemness of cancer cells, promote epithelial-mesenchymal transition (EMT) and invasion of surrounding tissues (Asif et al., 2021). CAFs remodel the ECM via secretion of ECM proteins and remodeling enzymes which stiffens the ECM, leading to chemoresistance via preventing the diffusion of anti-cancer drugs to the tumor tissue (Wei et al., 2022). CAFs also lead to an immunosuppressive microenvironment via changing the cellular repertoire in the TME (Zhang et al., 2023a). All these pro-tumorigenic functions of CAFs are summarized in Figure 2 and discussed below in detail.

### 4.1 The effects of cancer-associated fibroblasts on non-cellular components of the tumor microenvironment

In healthy tissues, the ECM is in a balance between continuous degradation and reconstruction to ensure its structural integrity and proper functioning. This process includes synthesizing new matrix proteins to replace the old ones. In most solid tumors, CAFs break this balance mainly by over-secreting diverse ECM proteins. Hence, CAFs lead to a significant increase in tissue and matrix stiffness. This transformation creates a supportive and conducive environment for tumor growth and invasion (Wei et al., 2022).

Tissue stiffness could be explained as the resistance of biological tissues to deformation, ranging from the soft and compliant nature of brain tissue to the stiffness and resistance to deformation seen in bone tissue (Wells, 2013). The stiffness of a tissue could be altered in a disease state. In desmoplastic tumors such as breast cancer, pancreas cancer, and prostate cancer, tumor tissue often exhibits an abnormally stiff structure compared to normal tissue (Ishihara and Haga, 2022). The critical component driving this re-regulation of tissue–matrix stiffness in these solid tumors is the increased expression of stiffness-promoting matrix components, such as collagen and fibronectin (FN), by CAFs (Asif et al., 2021; Ishihara and Haga, 2022).

CAFs produce high amounts of collagen, a major ECM component, resulting in the accumulation of collagen fibers within the ECM (Asif et al., 2021). CAFs also affect the organization and alignment of collagen fibers within the ECM via the secretion of enzymes such as lysyl oxidase (LOX), which form covalent bonds between collagen fibers, leading to cross-links (Winkler et al., 2020). Hence, ECM becomes more rigid and resistant to mechanical deformation (Setargew et al., 2021). Matrix stiffening through LOX-induced collagen crosslinking and increased focal adhesion formation in breast cancer led to increased invasion of premalignant mammary epithelial cells and the release of inflammatory cytokines (Levental et al., 2009). Similarly, in gastric cancer, CAF-induced LOX overexpression positively correlated with increased proliferation rate, migration, and invasion into surrounding tissue. Inhibition of LOX reduced these effects, which suggested LOX as a potential target for anti-stromal therapies (Li et al., 2015).

The second major component of ECM is fibronectin (FN). Fibronectin is a glycoprotein that functions in cell adhesion, migration, and wound healing (Patten and Wang, 2021). Fibronectin production by CAFs induces the development of fibrosis, a condition characterized by excess deposition of ECM components, eventually leading to increased tissue stiffness (Singh et al., 2010; Wynn and Ramalingam, 2012). Fibronectin can cross-link collagen and act as a scaffold and template for organizing collagen fibrils. This was confirmed by the incapability of the fibronectin-deficient mouse fibroblast cell line to form a collagen matrix despite an efficient expression of collagens. The transfection of these cells to express FN enhanced the matrix formation. Numerous studies also suggest that collagen can enhance FN assembly. For instance, chick embryo fibroblasts required the presence of collagen binding sites for the assembly of recombinant FNs. The ectopic expression of the collagen significantly increased the formation of the FN matrix in collagen type I deficient cells of mice. This intricate interplay between FN and collagen and their overexpression by CAFs ultimately contributes to an increase in matrix stiffness in cancer (Singh et al., 2010).

In many cancers, the levels of CAF infiltration and ECM stiffness correlate with tumor aggressiveness (Chen et al., 2020). Increased matrix stiffness in the TME alters many cellular processes and significantly impairs drug delivery and drug uptake in tumor tissue (Wang et al., 2017). The actions of conventional antineoplastic drugs are generally dose-dependent. Drugs should reach an effective concentration at the tumor site (Deng et al., 2022). However, stiffened ECM creates physical barriers that hinder the penetration of drugs into the tumor. Tumor cells residing deeper within the stiff matrix may be less accessible to the drug. Also, the increased stiffness alters the fluid dynamics by elevating the interstitial pressure, limiting the delivery of drugs from circulation to the tumor tissue. Hence, the stiff matrix compromises the efficacy of anti-cancer drugs. For instance, doxorubicin was shown to be the least effective in breast cancer models with the highest stiffness (Deng et al., 2022). Paclitaxel, and cisplatin also exhibited higher IC50 values in MCF-7 cells with stiffer ECM (Feng et al., 2013).

In addition to preventing drug delivery by creating a physical barrier, CAFs also contribute to the induction of cellular chemoresistance mechanisms. Cancer models with a stiffened matrix coated with FN demonstrated enhanced repair of cytotoxic drug-induced double-strand DNA breaks. This process is governed by the MAP4K4/6/7 kinase, which triggers the phosphorylation of ubiquitin. Then, phosphorylated ubiquitin induced the recruitment of H2AX to DNA damage sites, activating the DNA repair mechanisms (Deng et al., 2020). A recent study showed that CAFs increase the survival and decrease the apoptosis of gastric cancer cells upon 5-Fluorouracil (5-FU) treatment. RNA sequencing of CAFs has revealed that Neuropilin 2 (NRP2), a transmembrane glycoprotein that functions as VEGFR, was the highest transcript compared to normal fibroblasts (Yang et al., 2022). The study highlighted that resistance to 5-FU is associated with NRP2 and mediated by stromal-derived growth factor (SDF-1), also known as C-X-C motif chemokine 12 (CXCL12). Silencing NRP2 led to the downregulation of H2AX, hence the DNA damage repair. These suggest that CAFs not only support cancer cells through soluble mediators but may also act on receptor proteins to activate survival pathways in cancer cells.

### 4.2 The interaction of cancer-associated fibroblasts with immune cells in the tumor microenvironment

The interaction of CAFs with the immune cells within the highly interactive TME significantly facilitates tumor progression by creating an immunosuppressive milieu. CAFs alter both innate and adaptive immune cells, weakening immune responses against tumor cells. CAFs upregulate the immune checkpoint molecules and modify the ECM to regulate immune cells’ mobility and anti-tumor effects indirectly (Zhang et al., 2023a). In recent studies, particularly the interactions of CAFs with macrophages and T cells have emerged as prominent regulators of tumor immune microenvironment.

#### 4.2.1 Interaction between cancer-associated fibroblasts and tumor-associated macrophages

The macrophages are a part of the first line of immune defense, so-called innate immunity. In TME, macrophages are transformed into tumor-associated macrophages (TAMs), further augmenting the tumorigenic microenvironment (Lin et al., 2019). CAFs play essential roles in this transformation by releasing substantial levels of SDF-1, chemokine ligands (CXCLs), and IL-6, which recruit macrophages to the TME. CAFs isolated directly from breast tumor tissue of transgenic mice and cocultured with breast cancer cells exhibited a significant upregulation of pro-inflammatory factors: CXCL1, Chitinase-3-like-1 (Chi3L1), and IL-6. These CAFs significantly increased the migratory ability of macrophages when co-cultured (Cohen et al., 2017). CAF-derived M-CSF and IL-6 were shown to recruit and activate macrophages in pancreatic cancer (Gunaydin, 2021).

Just as CAFs play a role in their recruitment into TME, TAMs trigger CAF activation by secreting several growth factors, such as FGF, VEGF, and PDGF. TAMs also activate epithelial-mesenchymal transformation, which may foster the formation of new CAFs. Most importantly, upon the stimuli from CAFs, TAMs negatively regulate cytotoxic cells and promote the immunosuppressive regulatory T cells (Tregs) and myeloid-derived suppressor cells (MDSCs), inducing an immunosuppressive phenotype in TME (Gunaydin, 2021).

#### 4.2.2 Interaction between cancer-associated fibroblasts and T cells

T cells are the essential components of the adaptive immune system, playing critical roles in anti-tumor immunity in TME. CAFs contribute to an immunosuppressive microenvironment by inhibiting the activity of cytotoxic T cells while promoting the activity of Tregs. This hinders the immune system’s ability to effectively attack and eliminate cancer cells. Cytotoxic T cells, or CD8^+^ T cells, are especially crucial in targeting and destroying cancer cells. They induce apoptosis in tumor cells. These antitumor activities are alleviated by CAFs releasing various soluble factors that suppress the activity and function of cytotoxic T cells. For instance, TGF-β, IL-6, and prostaglandin E2 (PGE2), secreted by CAFs, are known to inhibit the proliferation and cytotoxic function of CD8^+^ T cells, leading to T cell exhaustion (Xie et al., 2021). CAFs were shown to induce the expression of PGE2, inhibiting the proliferation of CD8^+^ T cells by disrupting the cell cycle and downregulating the expression of key molecules required for T cell activation and proliferation (Finetti et al., 2020). Besides the direct inhibition of CD8^+^ T cells, CAFs also downregulate their recruitment into the TME via the secretion of chemokines like CXCL12 (Mezzapelle et al., 2022; Zhang et al., 2023a).

CAFs also promote the proliferation and recruitment of Tregs to the TME. The primary role of Tregs is suppressing cytotoxic T cells to provide self-tolerance and immune homeostasis (Kondělková et al., 2016). CAFs secrete cytokines, including TGF-β, IL-6, and IL-10, promote the generation, recruitment, or activation of Tregs in TME. TGF-β′s role in TME is two-fold; it promotes the activation of CAFs, and its secretion by CAFs induces the Treg accumulation in TME. Inhibiting TGF-β with gemcitabine in a mouse model of pancreatic cancer caused a significant decrease in the Treg/CD8+ T cell ratio (Koppensteiner et al., 2022). Inhibiting CAF activity using TGF-β blockers in urothelial cancer facilitated the infiltration of CD8^+^ T cells into the TME (Koppensteiner et al., 2022).

The use of immunotherapy has gained a great interest in the treatment of many cancer types. Immune checkpoint inhibitors are preferentially used to treat advanced-stage metastatic cancers such as melanoma (Emens and Middleton, 2015). Tumor cells or other TME cells express immune checkpoint ligands like PD-L1 that bind to PD-1 on immune cells and inhibit them. This interaction suppresses T cell function, preventing them from attacking the tumor. Immune checkpoint inhibitors disrupt this interaction of PD-1/PD-L1 and aim to avoid suppressing T cells (Emens and Middleton, 2015). However, CAFs contribute to resistance against immune checkpoint inhibitors. CAFs are known to be one of the sources of PD-L1 expression within the TME. The PD-L1 expressed on CAFs interacts with PD-1 on immune cells. This interaction effectively suppresses the immune response, limiting the ability of immune cells, particularly cytotoxic T cells, to attack and destroy cancer cells (Pei et al., 2023). Therefore, targeting CAFs may be crucial to overcome immunosuppression in the TME and enhance the efficacy of immunotherapies.

## 5 Significance of cancer-associated fibroblasts in gastric cancer

Gastric cancer is a highly heterogeneous disease with multiple subtypes. Histologically, the Lauren classification considers intestinal and diffuse types to be two main subtypes (Lauren, 1965). Intestinal-type gastric adenocarcinoma develops by the intestinalization of gastric mucosa and is comprised of malignant cohesive cells organized as glands. The diffuse type is characterized by undifferentiated cells with a non-cohesive phenotype infiltrating the gastric wall diffusely (Cisło et al., 2018). Diffuse-type gastric adenocarcinoma is considered more aggressive and chemoresistant, with an increased recurrence rate (Lin et al., 2015).

Endeavors to dissect the molecular mechanisms of gastric cancer enabled molecular classifications of this complex disease. The Cancer Genome Atlas (TCGA) initiative classified gastric tumors into four subtypes: the Eppstein-Bar Virus positive, microsatellite-instable (MSI), genomically stable tumors, and tumors with chromosomal instability (Cancer Genome Atlas Research Network, 2014). The Asian Cancer Research Group (ACRG) classified gastric cancers based on the correlation between molecular alterations and clinical presentation. ACRG classification associates the diffuse type with micro-satellite stable and epithelial-to-mesenchymal-transition (MSS/EMT) subtype, showing the worst prognosis and high recurrence rate. In contrast, the intestinal type is linked with the MSI subtype and better prognosis (Cristescu et al., 2015).

Recent studies highlight the significance of gene signatures and pathways for the prognostic classification of gastric cancer. Kim et al. identified two distinct molecular subtypes of diffuse-type gastric cancer: core diffuse-type (COD) and intestinal-type-like (INT). COD is associated with high recurrence, low survival rate, better response to chemotherapy, and increased expression of TGF-β and EMT-related genes. However, INT is linked with decreased levels of these genes, upregulation of DNA damage response genes, and better response to immune checkpoint inhibitors (Kim et al., 2020).

### 5.1 Tumor microenvironment and cancer-associated fibroblasts in the aggressiveness of gastric cancer

Understanding the effect of stroma and the immune microenvironment on the signaling pathways involved in gastric cancer progression is crucial for developing novel treatment strategies. Li and Wang proposed a pathway-based classification of gastric cancer, in which three subtypes are identified: tumors with immunity-deprived (ImD), immunity-enriched (ImE), and stroma-enriched (StE) microenvironment signature. Among these, StE is found to be the signature associated with the worst prognosis. The StE subtype is characterized by the overactivation of oncogenic pathways that drive tumor growth, progression, invasion, metastasis, and resistance to immunotherapy (Li and Wang, 2021).

Zeng et al. depicted the individual significance of TME elements for poor prognosis based on TME infiltration patterns, expression levels of stromal or immune-related signatures, and clinical data. They classified gastric TME into three distinct phenotypes and established a TME scoring system. CAF infiltration and upregulation of stromal-related genes emerged as key risk factors in the TME phenotype with the worst prognosis (Zeng et al., 2019). Our comprehensive analysis of extensive gastric cancer patient datasets revealed CAF infiltration and CAF markers as critical factors for poor prognosis (Ucaryilmaz Metin and Ozcan, 2022a).

Several studies further shed light on the role of CAFs in the aggressiveness of gastric cancer cells. CAF infiltration actively induced the progression of scirrhous type gastric cancer, an aggressive subtype of diffuse gastric adenocarcinoma (Miki et al., 2020). CAFs were shown to utilize soluble factors like FGF, TGF-β, cytokines, and ECM remodeling enzymes to stimulate the invasive capabilities of scirrhous-type gastric cancer cells. Chemokines produced by CAFs like CXCL12 also promote tumor progression and invasiveness of gastric cancer cells by clustering integrin β1 proteins on gastric cancer cells (Izumi et al., 2016). CAFs with high expression of CXCL2 were shown to correlate with dismal prognosis in gastric cancer patients (Qin et al., 2021).

Microarray-based bioinformatics studies revealed increased expression of CAF markers Fibronectin 1 (FN1), Serine proteinase inhibitor 1 (SERPINE1), and secreted protein acidic and cystine-rich (SPARC) genes in gastric cancer patients with low survival (Li et al., 2019). In a previous study, we identified six key CAF markers: collagen types COL3A1, COL1A2, COL1A1, COL5A1, and FN1, and SPARC as the pivotal poor prognostic markers in gastric cancer. The correlation of these markers with the CAF infiltration is found to be stronger than recently identified CAF markers like CXCL12 or TGF-β. In addition, we showed that these markers are highly expressed in gastric cancers with mesenchymal phenotype (Ucaryilmaz Metin and Ozcan, 2022a), which significantly contributes to the progression of the disease and poor overall survival (Yu et al., 2014; Huang et al., 2015).

Besides ECM proteins, soluble ligands secreted by CAFs can contribute to the aggressiveness of gastric cancer cells. Bae et al. show that CAFs secrete an AXL receptor ligand, GAS6, which leads to the phosphorylation of the AXL receptors on gastric cancer cells, increasing the viability and inducing EMT in gastric cancer cells. Silencing or inhibition of AXL reverted this phenotype and decreased the number of peritoneal nodules in a gastric cancer mouse model. High expression of phosphorylated AXL was associated with poor survival in gastric cancer patients (Bae et al., 2020).

### 5.2 The role of cancer-associated fibroblasts in gastric cancer metastasis

**FIGURE 3:**
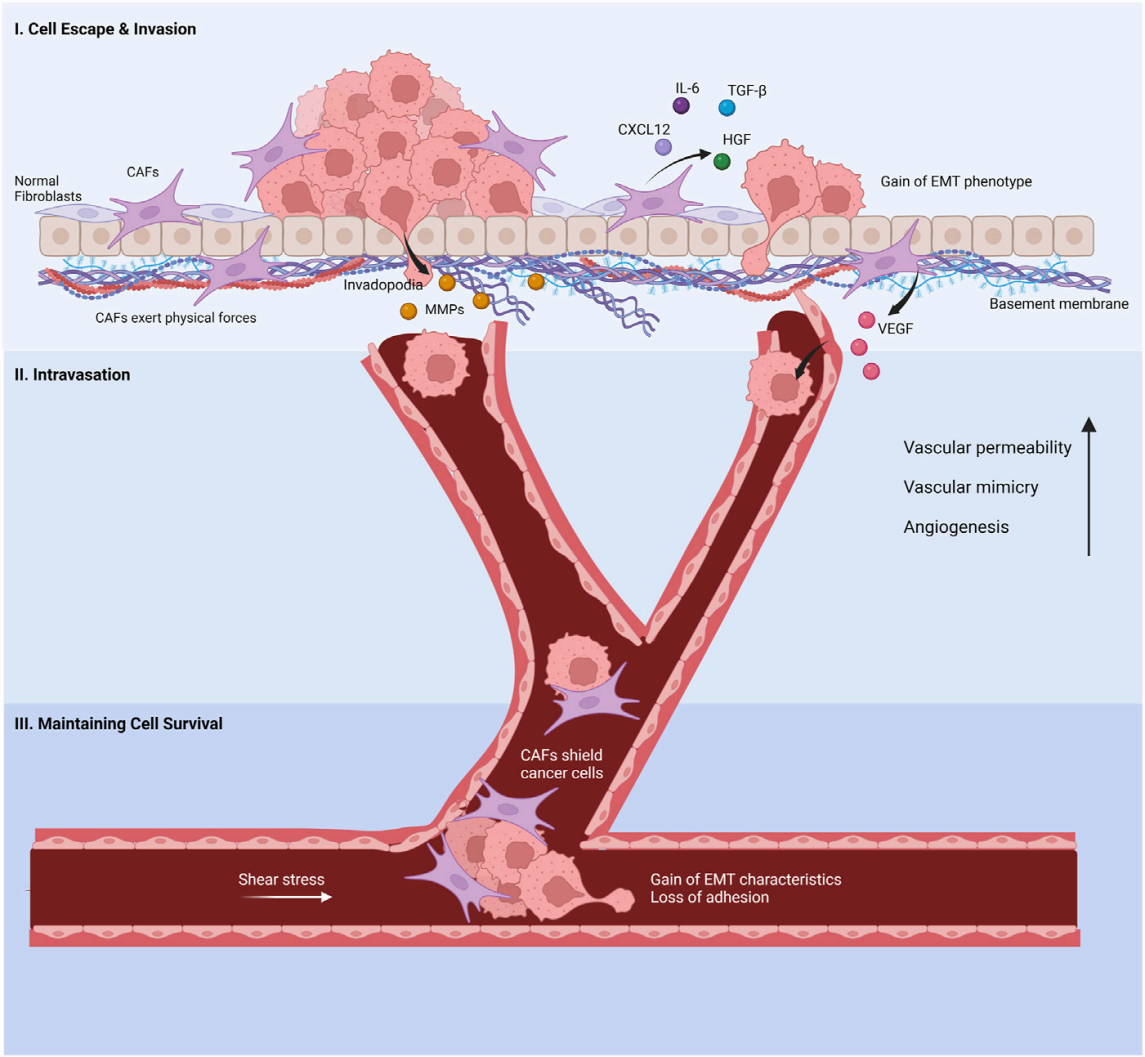
Cancer-associated fibroblasts’s involvement in the metastatic cascade. (I) Normal fibroblasts are activated by cancer cells to promote invasion. The cancer-associated fibroblasts (CAFs) help cancer cell evasion from the primary tumor site by secreting chemokines and proteins, inducing EMT phenotypes in cancer cells. Invasion through the basement membrane is aided by CAF-derived MMPs and physical forces that re-organize the ECM. (II) VEGF released by CAFs increases the leakage in capillaries, enabling cancer cells to enter the bloodstream. CAFs increase blood vessel generation and vascular mimicry to facilitate the intravasation of cancer cells. (III) CAFs protect cancer cells from shear stress and anoikis in the bloodstream. Created with BioRender.com.

Metastasis is a complex and multi-step process initiated at the tumor-stroma interfaces. Among many contributing factors, CAFs potently promote metastasis in gastric cancer as critical residents of the tumor stroma (Zhi et al., 2010). CAFs stimulate directly or indirectly the four steps of metastasis: I) evasion of cancer cells from the primary tumor site and invasion into surrounding tissue, II) intravasation, III) survival in the circulatory system, and IV) extravasation (Asif et al., 2021) (Figure 3).

#### 5.2.1 Cancer-associated fibroblasts promote cell escape and invasion

Metastasis starts with the evasion of cancer cells from the primary site of the tumor as single cells or multiple-cell clusters. Single cells escaping the tumor site lose cell-to-cell adhesion, downregulate E-cadherin expression, and gain EMT properties. Multi-cell clusters exhibit these properties partially since they need cellular contact to some extent (Majidpoor and Mortezaee, 2021). CAFs enhance the EMT of cancer cells, and hence invasion, through the synthesis of TGF-β protein and activation of TGF-β/SMAD pathway (Chandra Jena et al., 2021). A recent study suggests EMT in gastric cancer is also induced by the overexpression of Neuronal Regeneration Related Protein (NREP) through interaction with the TGF-β1. The study also indicates that CAF infiltration and recruitment correlate with NREP overexpression (Liu et al., 2021b). IL-6, CXCL12 and Hepatocyte growth factor (HGF) secreted by CAFs also contribute to EMT (Izumi et al., 2016; Wu et al., 2017; Chandra Jena et al., 2021).

Besides cytokines, CAFs secrete ECM remodeling and degrading enzymes that alter the physical forces in the ECM and facilitate the invasion of cancer cells (Glentis et al., 2017; Kwa et al., 2019). Matrix metalloproteinases (MMPs) that degrade ECM are activated at the outermost borders of invading cancer cells, forming invadopodium structures. These processes aid in disintegrating the barrier of the basement membrane, building an open field for the cancer cells to migrate (Glentis et al., 2017; Kwa et al., 2019). ECM crosslinking LOX-Like-2 enzyme, secreted by gastric CAFs, aids migration and invasion of gastric cancer cells through the FAK/Src pathway (Kasashima et al., 2014). Although the association of LOX with collagen fibers and its downstream effect are somewhat vague in gastric CAFs, recent evidence claimed that small extracellular vesicles secreted by oral squamous cell carcinoma (OSCC) CAFs, which contain active LOX, interact with fibronectin and periostin to bind to collagen fibers through integrin 2αβ1 (Liu et al., 2023). This interaction led to collagen crosslinking, which phosphorylated FAK/paxillin, causing activation of the Rho/ROCK pathway and subsequent actomyosin contraction, causing nuclear translocation of YAP to induce EMT of OSCC cells (Liu et al., 2023). In scirrhous gastric cancer, CAFs that are in direct contact with the cancer cells were shown to induce invasion by mechanically remodeling the ECM through actomyosin contraction (Yamaguchi et al., 2014).

Increased expression of transgelin, a protein involved in actin-mediated contraction, in CAFs was found to regulate the invasion of gastric cancer cells through upregulating MMP-2 production (Yu et al., 2013). Another study suggested that gastric CAFs remodel the ECM via the production of hyaluronan and proteoglycan link protein 1 (HAPLN1) and promote the invasion of gastric cancer cells (Zhang et al., 2022). The authors also showed that gastric cancer cells stimulate the HAPLN1 synthesis by CAFs by activating the TGF-β/SMAD signaling pathway. Increased HAPLN1 expression altered the collagen deposition by decreasing their length, width, and density in ECM. Thus, CAFs indirectly allowed gastric cancer cells to invade through the ECM. These results demonstrate the cooperation between CAFs and gastric cancer cells to remodel ECM and establish a favorable microenvironment for invasion. In contrast, overexpression of HAPLN1 in colorectal cancer cells led to a decrease in the migration of these cells and tumor size (Wang et al., 2021a). In colorectal cancer cells, reduced HAPLN1 levels were associated with the activation of the TGF-β/SMAD pathway and increased collagen production. This opposing interaction between cancer cells and CAF-derived HAPLN1 in different cancers may indicate the significance of the composition of all CAF secretomes for the invasion of cancer cells since other factors secreted by CAFs may determine in which direction the HAPLN will act.

Podoplanin, a transmembrane protein interacting with RhoA, is another mediator of CAF-induced gastric cancer invasion. Kondo and colleagues observed that podoplanin-expressing CAFs positively affected the invasion of both gastric signet ring cells and gastric tubular adenocarcinoma cells. However, silencing podoplanin did not affect the invasion of aggressive gastric signet ring cells despite decreasing the invasion of gastric tubular adenocarcinoma cells (Kondo et al., 2023). These findings suggested that aggressive gastric cancer cells may devise additional invasion-related pathways independent from CAFs.

#### 5.2.2 Cancer-associated fibroblasts promote angioneogenesis and vascular permeability

Entry of cancer cells into the blood or lymphatic system is central for their transport to distant sites. Vascularization and angiogenesis expedite this process (Chiang et al., 2016). CAFs act both in the induction of angiogenesis and vascular permeability, facilitating the entrance of cancer cells into the circulation. Transcriptome analysis in gastric cancer revealed that activated fibroblasts are correlated with angiogenesis and confirmed their association with poor prognosis (Oshi et al., 2021). CAFs produce one of the major angiogenic factors, VEGF, known as the vascular permeability factor, which causes hyperpermeability in vessels and increases microvessel density (Kitadai, 2010). In gastric cancer, CAFs induce an increase in the expression of VEGF and phosphorylated VEGF Receptor-2 (VEGFR-2) through Galectin-1, which is a carbohydrate-binding protein with tumor invasion and angiogenesis-promoting effects (He et al., 2014; Tang et al., 2016). Ding and colleagues point out the significance of CAF-derived HGF on the promotion of angiogenesis and Vasculogenic Mimicry (VM) of gastric cancer cells through the PI3K/AKT and ERK1/2 signaling axis (Ding et al., 2018). In gastric cancer, CAF-derived miR-29b-1-5p increased VM and migration through upregulating N-cadherin and vimentin (Wu et al., 2023). However, CAF-secreted miR-29b in hepatocellular carcinoma prevents VM by interfering with IL-6 signaling, inhibiting the STAT3 pathway and MMP-2 production (Fang et al., 2017). Therefore, CAF-derived miRNAs and their effect on the VM of cancer cells can differ in distinct cancers owing to the different characteristics of CAFs.

Hypoxic environments further enhance angiogenesis by upregulating proangiogenic factors and inflammatory mediators or growth factor signaling pathways (Ucaryilmaz Metin and Ozcan, 2022b). Park et al. suggested that hypoxic CAFs promote some of the gastric cancer cell lines’ migratory and invasion capabilities by downregulating COL4A2 expression (Park et al., 2023). However, there is no substantial evidence of how hypoxic CAFs induce these effects, and variations between cell lines imply the involvement of other mediators in this process.

#### 5.2.3 Cancer-associated fibroblasts contribute to the survival of Circulating Tumor Cells

Cancer cells that invade successfully into the blood or lymphatic vessels are called Circulating Tumor Cells (CTCs). Most CTCs do not survive in the stressful environment of circulation. To survive, they must adapt to oxidative stress and shear forces caused by the liquid flow, evade immune destruction, and avoid anchorage-dependent cell death anoikis. Recent findings suggest that CTCs travel through the circulatory system as cellular aggregates clinging to CAFs (Ortiz-Otero et al., 2020a; Sharma et al., 2021). Thus, CAFs protect CTCs from shear stress, enabling them to sustain their proliferative capability and facilitate migration.

A comprehensive study compared levels of circulating CAFs for nine different types of cancers, which revealed colon cancer to be the cancer with the highest number of circulating CAFs and gastric cancer to be the cancer with the lowest number of CAFs in the bloodstream (Ortiz-Otero et al., 2020b). However, in patients with metastatic gastric cancer, increased levels of circulating CAFs, but not CTCs, were correlated with poor prognosis. In these patients, circulating CAFs were observed together with CTCs as clusters, and 70% of the CTCs showed mixed epithelial/mesenchymal characteristics. The study also suggested that upon combinational therapy of 5-fluorouracil, irinotecan, and oxaliplatin, the levels of CTCs increase in the blood compared to baseline (Ortiz-Otero et al., 2020b). Therefore, discovering the interplay between CAFs and CTC release upon chemotherapy application may help us understand the underlying mechanisms of metastatic niche formation and drug resistance.

### 5.3 The role of cancer-associated fibroblasts in gastric cancer stemness

CSCs are tumor cells that hijack stem cells’ self-renewal, differentiation, and proliferative capabilities, enabling them to boost tumor progression, metastasis, chemotherapy resistance, and immunomodulation. CSCs are generated through the proliferation and differentiation of adult stem cells, which bear several mutations, oncogene activations, and tumor suppressor inactivation (Walcher et al., 2020). The induction of EMT through EMT-related transcription factors is considered a major driver of the CSC generation. In addition to EMT, TGF-β, Notch, Wnt, Hedgehog, NF-kB, and PI3K/AKT pathways are highly associated with CSC generation (Lim et al., 2021). Besides the upregulation of these pathways, variations in the cluster of differentiation (CD) molecules characterize CSCs in different types of cancers. In gastric cancer, CD44 variants, metabolic enzyme ALDH1, and essential stem cell markers Oct-4 and Sox2 are commonly observed, culminating in a tumorigenic and chemoresistant phenotype of CSCs (Bekaii-Saab and El-Rayes, 2017).

Recent findings suggest that both noncellular and cellular components of TME contribute to CSCs’ sustainability. CSCs overexpress ECM components like proteoglycans, polysaccharides, fibrous ECM proteins, glycoproteins, and their receptors to support their self-renewal and survival (Nallanthighal et al., 2019). CAFs further potentiate the stemness of CSCs through increased ECM protein production, matrix remodeling, paracrine signaling, and nutrient supply (Nallanthighal et al., 2019; Loh and Ma, 2021).

#### 5.3.1 Cancer-associated fibroblast-derived glycoproteins and stemness in gastric cancer

Most glycoproteins in the ECM enhance the stemness of cancer cells by binding to integrins and activating downstream pathways. For instance, periostin is a glycoprotein secreted by fibroblasts that promotes cross-linking of collagen I with other glycoproteins like FN and tenascin-C (Kudo and Kii, 2018). Periostin boosted the stemness, migration, and invasion of several cancers (Huang et al., 2023b). A recent study suggests that periostin secreted by podoplanin positive-CAFs induces metastasis and stemness in gastric cancer cells through PI3K/AKT and FAK/YAP activation, respectively. The study highlights that FAK/YAP signaling promotes IL-6 expression in gastric cancer cells, and IL-6 acts as a positive feedback loop to activate the PI3K/AKT pathway further to produce more periostin (Zhao et al., 2023a). This study confirmed that decreased periostin levels attenuate gastric cancer cells’ stemness properties, such as sphere formation. CAF-secreted neuregulin-1, a glycoprotein that binds to ERBB3 receptor tyrosine kinase, has also been shown to increase the self-renewal of gastric cancer cells through the NF-kB signaling pathway (Han et al., 2015).

Although some glycoproteins increase the stemness of several cancer cell types, SPARC glycoprotein was shown to have a cell type-specific role in regulating the stemness of different cancer types. For instance, high levels of SPARC induced stemness in endometrial and hepatocellular carcinomas but not in gastric cancer cells (Yusuf et al., 2014; Jiang et al., 2019). Ma and colleagues suggest that SPARC secreted by gastric CAFs is conversely related to the stemness of gastric cancer cells (Ma et al., 2019). However, more effort is required to unravel this discrepant role of SPARC in stemness.

#### 5.3.2 Cancer-associated fibroblasts induce stemness in gastric cancer through TGF-β signaling

CAF-induced TGF-β signaling promotes tumor progression not only by enhancing proliferation, invasion, or angiogenesis but also by stemness of cancer cells. The binding of TGF-β to TGF-β Receptor (TGF-βR) activates downstream pathways like the canonical TGF-β/SMAD pathway or PI3K/AKT or ERK pathways. Increasing evidence suggests that CAFs secrete TGF-β itself as well as other factors to boost TGF-β signaling (Fang et al., 2022). In scirrhous-type gastric cancer, CAFs were shown to upregulate the gastric CSC markers, CSC population, and their expression of TGF-βR1, TGF-βR2, and SMAD2 (Hasegawa et al., 2014). In this gastric cancer model, TGF-β neutralizing antibodies decreased the stemness of gastric CSCs. TGF-β also promoted CAFs to secrete Insulin-like Growth Factor Binding Protein-7 (IGFBP7) that augments the stemness of gastric cancer cells (Hong et al., 2023). These findings show that CAFs promote the generation, survival, and maintenance of CSCs in gastric cancer.

### 5.4 The role of cancer-associated fibroblasts in an immunosuppressive microenvironment in gastric cancer

Growing evidence indicates that CAFs induce an immunosuppressive microenvironment via activation of immune checkpoints and modulation of the infiltration and activity of immune cells in gastric cancer. A bioinformatic analysis of the tumor samples from TCGA-stomach adenocarcinoma revealed that high CAF infiltration was associated with increased infiltration of M2 macrophages (Gao et al., 2023), which induces an immunosuppressive environment, angiogenesis, and tumor growth (Jayasingam et al., 2020). High CAF infiltration was also associated with a poor response to immunotherapy in gastric cancer patients (Gao et al., 2023). Another analysis of gastric cancer patient cohorts confirmed these results and suggested that high CAF infiltration is also associated with decreased infiltration of NK cells but not cytotoxic T cells (Lu et al., 2022). Liu et al. observed that CAF infiltration was negatively correlated with T-helper cells and positively correlated with monocytes, dendritic cells, and M2 macrophages in gastric cancer cohorts. The authors also detected ten different checkpoints, including PD-1 and PD-L2, which were upregulated in gastric cancers with high CAF infiltration (Liu et al., 2021a).

Recent studies dissect the genes involved in the induction of an immunosuppressive TME by CAFs. An *in vitro* study using CAF and gastric cancer cell co-culture models suggested that IL-8 secreted by CAFs induces the PD-L1 expression via activating JNK, P38, and NF-κB signaling pathways. Inhibition of IL-8 receptor CXCR1/2 effectively reduced the expression of PD-L1 in gastric cancer cells (Lou et al., 2023). This study may suggest that cytokines such as IL-8 secreted by CAFs can increase the expression of immunosuppressive ligands on cancer cells, leading to an immunosuppressive microenvironment in gastric cancer. However, the findings should be validated *in vivo* models and patient cohorts since *in vitro* models do not mimic the complexity of the tumor microenvironment.

Gu et al. established a prognostic CAF risk score with three genes, namely, GLT8D2 (glycosyltransferase eight domain containing 2) and CAF markers SPARC and VCAN. Patients with high CAF risk scores poorly responded to immunotherapy (Gu et al., 2023). In a single-cell transcriptomics analysis, SERPINE2 secreted by CAFs emerged as the key marker for an immunosuppressive and pro-tumorigenic microenvironment in gastric cancer patients (Zhang et al., 2023b). Although these studies provide insight into the markers of immunosuppressive TME in gastric cancer, how these markers induce an immunosuppressive environment is unknown. Mechanistic studies in models representing the complexity of TME as much as possible can shed light on these mechanistic links and reveal new therapeutic targets.

### 5.5 The role of cancer-associated fibroblasts in gastric cancer chemoresistance

Cancer cells can be resistant to anti-cancer drugs intrinsically or acquire resistance after drug exposure. Chemoresistance in gastric cancer is the most significant handicap in therapy, leading to high recurrence and very low survival rates. Although tumor cells integrate different mechanisms for developing resistance, the tumor microenvironment further potentiates the resistant phenotype of cancer cells. In particular, CAFs have a significant role in promoting the drug-resistant phenotype of cancer cells through paracrine mediators, exosomes, and ECM proteins. Gastric cancer is one of the cancers where CAFs play significant roles in drug resistance (De et al., 2022).

#### 5.5.1 Cancer-associated fibroblasts-derived interleukins (ILs) in gastric cancer chemoresistance

Interleukins are cytokines that regulate immunological processes as well as cancer progression. They contribute to tumorigenesis by activating protumorigenic pathways that augment cancer cells’ survival, invasion, stemness, or chemoresistance. IL-6 is known to be produced by cancer, immune, and stromal cells and is considered a significant player in cancer chemoresistance. A comprehensive study revealed that CAF-derived IL-6 causes resistance to 5-FU *in vitro* and *in vivo* models of gastric cancer. IL-6 exerted its effect by phosphorylating JAK1/STAT3, which increased cell survival and decreased apoptosis. The expression of IL-6 positively correlated with the stroma-related gene signatures of patients non-responsive to chemotherapy (Ham et al., 2019). However, the downstream effects of the JAK1/STAT3 axis and its relevance to 5-FU resistance in gastric cancer need further delineation.

In patients with advanced gastric cancer who receive platinum-based chemotherapy, serum IL-8, but not IL-6 level, was associated with poor clinical outcomes. IL-8 was found to be present in chemo-resistant patients before neoadjuvant therapy, and CAFs isolated from these patients showed increased levels of IL-8 secretion. Exogenous application of IL-8 on gastric cancer cells increased cell survival upon cisplatin treatment. It was demonstrated that IL-8 exerts its effect through NF-kB and PI3K/AKT axis. Moreover, high levels of IL-8 correlated with increased levels of drug transport protein ATP-binding cassette subfamily B member 1 (ABCB1), which is a key mediator of chemoresistance through decreasing the intracellular concentration of anti-cancer drugs (Zhai et al., 2019).

Another study supported that oxaliplatin resistance in gastric cancer is mediated by CAF-derived IL-8. Like the findings of Zhai et al. (2019), this action was through the activation of the CXCR2 and PI3K/AKT axes (Zhao et al., 2023b). Interestingly, the chemoresistance induced by CAFs in gastric cancer cells is reversed by activating the Vitamin D receptor (VDR) with a Vitamin D analog, Calcipotriol (Zhao et al., 2023b). Although the effect of VDR on cancer is known to some extent in the literature, the interplay between vitamin D and CAFs is new. Further studies on this interplay may increase the interest in vitamin D in cancer treatment.

IL-11, a member of the IL-6 cytokine family, is also known for its pro-tumorigenic role. Evidence suggests that IL-11 secreted by CAFs increases the survival of gastric cancer cells and promotes resistance to cisplatin, doxorubicin, and etoposide. This IL-11-mediated drug resistance is found to be through the JAK/STAT3 pathway, which results in higher expression of the anti-apoptotic gene Bcl-2. Blocking JAK by Ruxolitinib reversed chemoresistance and increased survival in the study (Ma et al., 2018). Therefore, inhibition of downstream effectors of interleukins may be promising to overcome CAF-induced chemoresistance in gastric cancer.

#### 5.5.2 Cancer-associated fibroblasts-derived growth factors in gastric cancer chemoresistance

Growth factor signaling pathways can also mediate CAF-induced resistance to cancer therapy. A study in HER2+ metastatic gastric cancer revealed that resistance to Trastuzumab could be surpassed by combining it with tyrosine kinase inhibitor Lapatinib, which targets the ATP-binding region of HER2. However, the study highlighted that using tyrosine kinase inhibitors can result in acquired resistance due to the activation of other receptor kinases. Surprisingly, in this study, CAFs have been shown to mediate this acquired resistance to Trastuzumab/Lapatinib combination. In Lapatinib-resistant tumor sections, phosphorylation of the Mesenchymal-epithelial-transition (MET) receptor, a receptor tyrosine kinase for HGF, was upregulated. CAFs isolated from these tissues exhibited an abundance of HGF. Therefore, the study concludes that CAF-derived HGF induces MET activation, which contributes to developing resistance to drugs that target HER2 (Ughetto et al., 2021).

Although CAF-derived growth factors contribute to chemoresistance, growth factor binding proteins exhibited an opposite role in lung cancer. Remsin Rix and colleagues showed that IGF secreted by CAFs promoted drug resistance in lung cancer through binding IGF-receptor (IGFR). However, CAF-secreted IGF-binding proteins (IGFBPs) resulted in drug sensitization by preventing IGFR-mediated FAK signaling (Remsing Rix et al., 2022). The authors found that either the action of IGF or IGFBPs dominated the CAF-lung cancer cell cocultures in a cell-type-dependent manner. Hence, IGF signaling resulted either in chemoresistance or chemosensitivity in a context-dependent manner. These results point out the significance of delineating the secretome of CAFs in specific contexts, which depends on the bidirectional communication between CAFs and cancer cells. Similar context-dependent action of CAF-induced growth factor signaling can also be valid for gastric cancer. However, this hypothesis warrants testing in gastric cancer.

#### 5.5.3 Cancer-associated fibroblasts mediate tissue stiffness-induced chemoresistance in gastric cancer

Cancer cells induce stromal cells to organize and produce ECM components. As a result, ECM crosslinking and stiffness are increased in cancerous tissues. Increased tissue stiffness is sensed by both cancer and stromal cells, resulting in further promotion of tumor progression by activating more fibroblasts and increasing the ECM protein production (Wei et al., 2022). The dense ECM acts as a barrier for drugs to penetrate and decreases drug delivery. Therefore, enhanced tissue stiffness is highly correlated with drug resistance in cancer (Jena et al., 2020).

Recently, a single-cell RNA sequencing study found that microfibril-associated protein-2 (MFAP2), an ECM glycoprotein that mainly functions to control ECM deposition, is associated with chemoresistance in cancer (Wei et al., 2023). The MFAP2 was primarily expressed in fibroblast populations and correlated with CAF markers in gastric tumor tissue. The study highlights that MFAP2-positive CAFs can be used as a prognostic marker for poor survival and resistance to adjuvant chemo/chemoradiotherapy and immunotherapy in gastric cancer. Also, the functional gene enrichment analysis revealed the association of MFAP2+ CAFs with the ECM organization and type I collagen synthesis. These findings suggested that MFAP2+ CAFs may limit drug delivery due to excessive crosslinking of ECM components in gastric cancer.

Hyaluronan, a glycosaminoglycan, is one of the major ECM polymers secreted by CAFs that provides elasticity and is involved in many signal-relaying pathways that drive cell proliferation, migration, and survival of cancer cells (Kobayashi et al., 2020). An increased hyaluronan-mediated motility receptor (HMMR), a hyaluronan receptor, predicted poor survival, resistance to 5-FU, and increased stemness in gastric cancer (Zhang et al., 2019). CD147, later renamed to EMMPRIN, is a cell surface transmembrane protein that affects hyaluronan production, its downstream effectors, and multi-drug resistance. Wang and colleagues showed that silencing of CD147 in gastric cancer cells decreased the sensitivity to cisplatin, invasion, and proliferation (Wang et al., 2010).

## 6 Heterogeneity in cancer-associated fibroblasts and single-cell sequencing studies

Recent studies reveal that CAFs vary in their characteristics in different tissues and in different niches of the same tumor, which leads to different functions (Jeong et al., 2021). A state-of-the-art technology, single-cell RNA-sequencing (scRNA-seq), enables us to dissect the distinct properties of CAF subtypes, investigate their spatial differences, and characterize their molecular functions. A scRNA-seq analysis of 768 mesenchymal cells in a mouse model of breast cancer exhibited three central populations of CAFs: vascular, matrix, and developmental CAFs (Bartoschek et al., 2018). These three subtypes differed in terms of their precursors and gene expression profiles. Vascular CAFs originated from the perivascular region enriched for genes involved in vasculogenesis. Matrix CAFs emerged from residing fibroblasts and are enriched for genes associated with ECM and EMT. Developmental CAFs result from cancer cells undergoing EMT (Bartoschek et al., 2018). Each subtype represented a distinguishable secretory profile. For instance, matrix CAFs were associated with elevated ECM production, whereas developmental CAFs were characterized by their involvement in paracrine signaling. Thus, each CAF subtype contributes to disease development differently.

Subsequent scRNA-seq studies gave rise to a more functionally oriented classification of CAF subtypes. scRNA-seq studies in mouse models revealed three CAF subtypes common to triple-negative breast cancer and pancreatic cancer: inflammatory (iCAF), myofibroblastic (myCAF), and antigen-presenting (apCAF) CAFs. iCAFs expressed high levels of cytokines, myCAFs highly expressed contractile proteins, and apCAFs expressed MHC class-II associated genes with possible roles in immunomodulation (Elyada et al., 2019; Sebastian et al., 2020). The presence of these subtypes was also demonstrated in tumor samples of pancreatic ductal adenocarcinoma patients (Hu et al., 2022).

Despite these commonalities in the subclasses of CAFs in breast cancer and pancreatic cancer, a scRNA-seq study by Kim et al. led to a slightly different classification of CAF subtypes in gastric cancer. The authors discovered three subtypes of CAFs that influence the progression of gastric cancer in different ways: inflammatory (iCAF), myofibroblast (myCAF), and intermediate CAFs (inCAF), which have a medium gene expression profile between iCAFs and myCAFs. The study found that iCAFs were associated with increased invasion and stemness and were more common in diffuse-type gastric cancer compared to intestinal-type. iCAFs had a high expression of chemokines (CXCL1, CXCL2, CXCL5, CXCL6), interleukins (IL6, IL11, and IL24), and MMPs (MMP1, MMP3, and MMP10), with an upregulation of pathways associated with stemness, such as TNF, NF-kappa B, and cytokine signaling pathways. Since the study’s primary aim was not the profiling of CAF subtypes in gastric cancer, the data presented on other CAF subtypes were limited (Kim et al., 2022). Li et al. aimed to characterize CAF subtypes and their interaction with other TME components in gastric cancer. Their single-cell analysis of eight gastric cancer and adjacent mucosa of patient samples revealed two prominent subtypes of CAFs: iCAFs and extracellular matrix CAFs (eCAFs). Both subtypes were correlated with an invasive phenotype and immunosuppressive TME in gastric cancer. The study showed high expression of IL-6 and CXCL12 in iCAFs and revealed their communication with T-cells. On the other hand, eCAFs highly expressed periostin, recruiting M2-like TAMs and enabling a favorable environment for metastasis. (Li et al., 2022). Another study focusing on the spatial distribution of different cell types in gastric cancer showed that fibroblasts with a gene expression profile similar to iCAFs were mainly localized at diffuse-type gastric cancer’s deep invasive layers (Jeong et al., 2021). Despite these significant findings, the number of gastric cancer samples investigated in scRNA-seq studies was limited. This may explain the differences in the classification of CAF subtypes and their characteristics in different studies. Larger scale scRNA-seq studies are needed to delineate the differences in the gene expression profile and function of distinct CAF subtypes.

The functional role of CAF subtypes also shows differences in distinct gastrointestinal cancers (Lavie et al., 2022). In pancreas cancer, a study revealed the immune protective role of iCAF due to enrichment in cytokine-cytokine receptor interaction and better prognosis (Hu et al., 2022). In contrast, gastric iCAFs were associated with dismal prognosis due to the upregulation of tumor-driving cytokine IL-6 and CXCL1/2 chemokines (Kim et al., 2022). Recent evidence highlights that, for colorectal cancer patients with elevated levels of immune-related-CAF infiltration, traditional chemotherapeutics could be more promising than immunotherapy applications since CAFs hijack their everyday functions (Zheng et al., 2021). Thus, CAFs not only act as stromal cells but rather like pseudo-immune regulatory cells due to their modulatory effect and rigorous communication with immune cells. However, these modes of communication with immune cells can be different in distinct cancers.

## 7 Therapeutic targeting of cancer-associated fibroblasts

Growing endeavors investigate effective strategies to prevent the action of CAFs in cancer. Among the vastness of the studies in the literature, here we review strategies that reached clinical trials. CAF-targeted therapies that aim to prevent the activation of fibroblasts or the pathways activated by CAFs dominate these studies.

The studies to prevent CAF activation focused on targeting CAF markers responsible for fibroblast activation, like FAP. Several preclinical studies showed that the inhibition or downregulation of FAP decreases tumor progression (Teichgräber et al., 2015; Dong et al., 2018), raising the interest in developing anti-FAP therapies. One of the first anti-FAP drugs, the anti-FAP antibody Sibrotuzumab (also known as F19 and BIBH 1) exhibited a good safety profile in patients with FAP-positive advanced or metastatic cancers in a Phase I trial (Scott et al., 2003). However, the study outcomes in terms of efficacy could not be met to continue the further clinical investigation in a Phase II trial in metastatic colorectal cancer (Hofheinz et al., 2003). Simlukafusp alfa, a biological agent which consists of a FAP binding site and IL-2 variant, has been developed to direct this immune cytokine to fibroblast-rich tumor tissue. Simlukafusp alfa increased the proliferation of NK cells and T cells in a dose-dependent manner *in vitro*. It improved the anti-cancer efficacy of monoclonal antibodies and immune checkpoint inhibitors *in vivo* models (Waldhauer et al., 2021). Furthermore, a phase II trial showed anti-tumor activity and tolerability of simlukafusp alfa with immune checkpoint inhibitor atezolizumab in metastatic cervical cancer patients (Italiano et al., 2021). However, the sponsor ceased further exploration of the drug because of a change in priorities in the portfolio (ClinicalTrials.gov, ID: NCT03386721). Nonetheless, several active trials test the use of FAP to direct imaging agents (Chandekar et al., 2023) and radiotheranostics (Privé et al., 2023) to the tumors with fibroblast-rich stroma.

FAP is also being tested as a target to direct CAR-T cells and other immunotherapy agents to tumor tissue. A phase I study reported a good safety profile for autologous anti-FAP CAR T-cells administered into the pleura of malignant mesothelioma patients (NCT01722149) (Curioni et al., 2019). NG-641 is an adenoviral vector which leads to the production of a bi-specific T cell activator that recognizes FAP (FAP-TAc). The expression of FAP-TAc induced by infection with NG-641 leads to the activation of T-cells and the killing of CAFs. The vector also encodes CXCL9, CXCL10, and IFN-α to recruit T cells (Champion et al., 2019). Several clinical trials are now actively investigating the safety profile of NG-641 as monotherapy (ClinicalTrials.gov, ID: NCT04053283) or in combination with immune checkpoint inhibitors in advanced cancers (Lillie et al., 2022; ClinicalTrials.gov, ID: NCT05043714; ClinicalTrials.gov, ID: NCT04830592).

Inhibiting CAF activation/recruitment or protumorigenic mediators released by CAFs is another strategy for targeting CAFs in cancer. A study utilized AMD3100 (also known as Plerixafor, which is clinically used in the treatment of leukemia for stabilizing hematopoietic stem cells) as an antagonist of CXCR4 against CAF-derived CXCL12/CXCR4 mediated signaling that facilitates invasion of cancer cells (Izumi et al., 2016). Thus, the antagonist prevented the stabilization of integrin αβ1 clustering in gastric cancer cells, inhibiting phosphorylation of focal adhesion kinases (FAK) and its adaptor protein, paxillin, decreasing the invasion of gastric cancer cells. Also, the study evaluated the effect of a FAK inhibitor PF-573228 to prevent gastric cancer cell invasion. Although FAK inhibitors attenuated the FAK phosphorylation directly, their impact on the invasion of gastric cancer cells was limited compared to Plerixafor (Wells, 2013). A phase I study investigated the effect of Plerixafor on immune therapy resistance in advanced-stage ovarian and colorectal cancers, with promising results (ClinicalTrials.gov, ID: NCT02179970; Biasci et al., 2020). However, no clinical studies focused on Plerixafor’s impact on gastric cancer.

Besides targeting CAF markers or CAF-induced signaling, preventing the development of CAFs may be an effective strategy. As we proposed in our previous study, halofuginone may be used as a promising agent in limiting CAF-driven cancer (Ucaryilmaz Metin and Ozcan, 2022b) since it prevents activation of myofibroblasts through inhibiting TGF-β/Smad3 pathway and limits tumor growth (Pines, 2014). A Phase I study tested halofuginone’s safety in advanced solid tumors and determined the dose for Phase II studies with the rationale that halofuginone exhibits antiproliferative, anti-metastatic, and anti-angiogenic action in preclinical models (de Jonge et al., 2006). Later, a Phase II study tested the effect of local halofuginone application to decrease the growth of human immunodeficiency virus (HIV)-related Kaposi’s sarcoma due to anti-angiogenic action (ClinicalTrials.gov, ID: NCT00064142). However, these studies are not focused on the anti-CAF action of halofuginone but rather its antiproliferative and anti-angiogenic effects. Further studies on the anti-CAF action of halofuginone may pave the way for its translation into clinical trials with a CAF-targeting focus.

Preclinical studies suggest that reverting the CAF phenotype to a senescent state via the inactivation of pluripotent stem cells and remodeling ECM may be other effective strategies (Chen and Song, 2019). Targeting EMT and hypoxia were also proposed as CAF-focused treatment strategies in the literature (De et al., 2022). However, these strategies have not yet been translated into clinical studies with a specific mention of anti-CAF action. Moreover, to our knowledge, a clinical trial that tests an anti-CAF strategy specifically in gastric cancer has not yet been registered into ClinicalTrials.gov. Further understanding of the CAF biology in gastric cancer may expedite this process.

## 8 Discussion and perspectives

Dynamic interactions between TME and cancer cells promote stemness, metastasis, and chemoresistance, leading to tumor progression and treatment failure in cancer. CAFs are the protagonists of TME in gastric cancer, associated with chemoresistance and dismal prognosis. Therefore, CAFs hold great promise as a potential therapeutic target in treating gastric cancer (Liu et al., 2021a; Lu et al., 2022). Despite the promise of CAF-targeting strategies in cancer, the lack of a complete understanding of the CAFs and their interaction with cancer cells may pose challenges in drug discovery.

The fact that CAFs derive from multiple cell types in the TME is one of the challenges for the therapeutic targeting CAFs. Moreover, the knowledge of the predominant cell lineage that gives rise to CAFs in specific cancers is incomplete. Current evidence indicates that different activation mechanisms operate in the differentiation of distinct cell types to CAFs (Ping et al., 2021). Hence, targeting a single lineage of differentiation may not be effective in blocking CAF formation since CAFs originating from multiple cell types may coexist in a TME. Another challenge is the heterogeneity of the resulting CAFs (Jeong et al., 2021). It is unclear whether the multiple origins of CAFs lead to this heterogeneity. However, further characterization of the CAF subtypes with larger-scale single-cell transcriptomics studies is crucial in gastric cancer.

The change of CAF phenotype with the changing TME further augments the challenge to target CAFs. The CAFs co-evolve with the dynamically changing TME, transitioning between quiescent fibroblasts, CAFs, and senescent CAFs, with different characteristics (D’Arcangelo et al., 2020). For instance, aging TME develops alterations with a predominant expression of fibroblast-associated genes and fibrotic changes. These alterations can change CAF phenotype, facilitate metastasis, and decrease response to therapy in cancer cells (Turrell et al., 2023). Despite growing awareness of the dynamic evolution of CAFs (Zhou et al., 2022), the secretory profile and modulatory role of CAFs in different stages of their life cycle are not fully characterized yet. Dissecting these changes in CAF phenotype is needed to guide CAF-targeting approaches effectively.

Due to varying phenotypes, one bullet-for-all approach may fall short of targeting CAFs in cancer. Accordingly, this may explain the ineffectiveness of some anti-FAP strategies tested in clinical trials (Hofheinz et al., 2003). Therefore, further characterization of different CAF subtypes and detailed identification of the markers at each subtype are essential. Although CAFs are mostly known for their tumor-promoting phenotypes, some subsets of CAFs can also function to restrain tumor progression in specific cancer types. An anti-tumorigenic secretome may explain this action of CAFs. For instance, Meflin, a tumor suppressor protein, was observed in low α-SMA expressing CAFs and inhibited pancreatic cancer progression (Miyai et al., 2020). Caveloin-1, CAF-derived mi-RNAs, and myofibroblastic CAF-secreted collagen type I are also proposed as CAF-derived tumor-suppressing elements in other cancers (Bhattacharjee et al., 2021; Wang et al., 2021). These studies point out that the secretome of CAFs is a key determinator of whether a CAF promotes the tumor or exhibits a tumor-suppressing effect. Therefore, a detailed mapping of the secretome of tumor-promoting vs tumor-suppressing CAFs may lead to identifying strategies that selectively target tumor-promoting CAFs.

## References

[B1] Aoto K. Ito K. Aoki S. (2018). Complex formation between platelet-derived growth factor receptor β and transforming growth factor β receptor regulates the differentiation of mesenchymal stem cells into cancer-associated fibroblasts. Oncotarget 9, 34090–34102. 10.18632/oncotarget.26124 30344924 PMC6183337

[B2] Arandkar S. Furth N. Elisha Y. Nataraj N. B. van der Kuip H. Yarden Y. (2018). Altered p53 functionality in cancer-associated fibroblasts contributes to their cancer-supporting features. Proc. Natl. Acad. Sci. 115, 6410–6415. 10.1073/pnas.1719076115 29866855 PMC6016816

[B3] Asif P. J. Longobardi C. Hahne M. Medema J. P. (2021). The role of cancer-associated fibroblasts in cancer invasion and metastasis. Cancers 13, 4720. 10.3390/cancers13184720 34572947 PMC8472587

[B4] Bae C. A. Ham I. H. Oh H. J. Lee D. Woo J. Son S. Y. (2020). Inhibiting the GAS6/AXL axis suppresses tumor progression by blocking the interaction between cancer-associated fibroblasts and cancer cells in gastric carcinoma. Gastric cancer official J. Int. Gastric Cancer Assoc. Jpn. Gastric Cancer Assoc. 23 (5), 824–836. 10.1007/s10120-020-01066-4 32239298

[B5] Bartoschek M. Oskolkov N. Bocci M. Lövrot J. Larsson C. Sommarin M. (2018). Spatially and functionally distinct subclasses of breast cancer-associated fibroblasts revealed by single cell RNA sequencing. Nat. Commun. 9, 5150. 10.1038/s41467-018-07582-3 30514914 PMC6279758

[B6] Baulida J. (2017). Epithelial-to-mesenchymal transition transcription factors in cancer-associated fibroblasts. Mol. Oncol. 11 (7), 847–859. 10.1002/1878-0261.12080 28544627 PMC5496490

[B7] Bekaii-Saab T. El-Rayes B. (2017). Identifying and targeting cancer stem cells in the treatment of gastric cancer. Cancer 123, 1303–1312. 10.1002/cncr.30538 28117883 PMC5412889

[B8] Belhabib I. Zaghdoudi S. Lac C. Bousquet C. Jean C. (2021). Extracellular matrices and cancer-associated fibroblasts: targets for cancer diagnosis and therapy? Cancers 13, 3466. 10.3390/cancers13143466 34298680 PMC8303391

[B9] Bhattacharjee S. Hamberger F. Ravichandra A. Miller M. Nair A. Affo S. (2021). Tumor restriction by type I collagen opposes tumor-promoting effects of cancer-associated fibroblasts. J. Clin. Investig. 131, e146987. 10.1172/JCI146987 33905375 PMC8159701

[B10] Biasci D. Smoragiewicz M. Connell C. M. Wang Z. Gao Y. Thaventhiran J. E. D. (2020). CXCR4 inhibition in human pancreatic and colorectal cancers induces an integrated immune response. Proc. Natl. Acad. Sci. U. S. A. 117 (46), 28960–28970. 10.1073/pnas.2013644117 33127761 PMC7682333

[B11] Cancer Genome Atlas Research Network (2014). Comprehensive molecular characterization of gastric adenocarcinoma. Nature 513, 202–209. 10.1038/nature13480 25079317 PMC4170219

[B12] Champion B. R. Besneux M. Patsalidou M. Silva A. Zonca M. Marino N. (2019). Abstract 5013: NG-641: an oncolytic T-SIGn virus targeting cancer-associated fibroblasts in the stromal microenvironment of human carcinomas. Cancer Res. 79 (13_Suppl. ment), 5013. 10.1158/1538-7445.Am2019-5013

[B13] Chandekar K. R. Prashanth A. Vinjamuri S. Kumar R. (2023). FAPI PET/CT imaging-an updated review. Diagn. Basel, Switz. 13 (12), 2018. 10.3390/diagnostics13122018 PMC10297281 37370912

[B14] Chandra Jena B. Sarkar S. Rout L. Mandal M. (2021). The transformation of cancer-associated fibroblasts: current perspectives on the role of TGF-β in CAF mediated tumor progression and therapeutic resistance. Cancer Lett. 520, 222–232. 10.1016/j.canlet.2021.08.002 34363903

[B15] Chen R. Huang L. Hu K. (2020). Natural products remodel cancer-associated fibroblasts in desmoplastic tumors. Acta Pharm. Sin. B 10, 2140–2155. 10.1016/j.apsb.2020.04.005 33304782 PMC7714988

[B16] Chen X. Song E. (2019). Turning foes to friends: targeting cancer-associated fibroblasts. Nat. Rev. Drug Discov. 18 (2), 99–115. 10.1038/s41573-018-0004-1 30470818

[B17] Chiang S. P. H. Cabrera R. M. Segall J. E. (2016). Tumor cell intravasation. Am. J. Physiol. Cell. Physiol. 311, C1-C14–C14. 10.1152/ajpcell.00238.2015 27076614 PMC4967137

[B18] Cisło M. Filip A. A. Offerhaus G. J. A. Ciseł B. Rawicz-Pruszyński K. Skierucha M. (2018). Distinct molecular subtypes of gastric cancer: from Laurén to molecular pathology. Oncotarget 9, 19427–19442. 10.18632/oncotarget.24827 29721214 PMC5922408

[B19] ClinicalTrials.gov, ID: NCT03386721 (2023a). Available at: https://clinicaltrials.gov/study/NCT03386721 (Accessed January 11, 2024).

[B20] ClinicalTrials.gov, ID: NCT04053283 (2023). Available at: https://clinicaltrials.gov/study/NCT04053283 (Accessed January 11, 2024).

[B21] ClinicalTrials.gov, ID: NCT00064142 (2024). Available at: https://clinicaltrials.gov/study/NCT00064142 (Accessed January 11, 2024).

[B22] ClinicalTrials.gov, ID: NCT02179970. Available at: https://clinicaltrials.gov/study/NCT02179970 (Accessed January 11, 2024).

[B177] ClinicalTrials.gov, ID: NCT04830592. Available at: https://clinicaltrials.gov/study/NCT04830592 (Accessed January 11, 2024).

[B178] ClinicalTrials.gov, ID: NCT05043714. Available at: https://clinicaltrials.gov/study/NCT05043714 (Accessed January 11, 2024).

[B23] Cohen N. Shani O. Raz Y. Sharon Y. Hoffman D. Abramovitz L. (2017). Fibroblasts drive an immunosuppressive and growth-promoting microenvironment in breast cancer via secretion of Chitinase 3-like 1. Oncogene 36, 4457–4468. 10.1038/onc.2017.65 28368410 PMC5507301

[B24] Cristescu R. Lee J. Nebozhyn M. Kim K.-M. Ting J. C. Wong S. S. (2015). Molecular analysis of gastric cancer identifies subtypes associated with distinct clinical outcomes. Nat. Med. 21, 449–456. 10.1038/nm.3850 25894828

[B25] Curioni A. Britschgi C. Hiltbrunner S. Bankel L. Gulati P. Weder W. (2019). A phase I clinical trial of malignant pleural mesothelioma treated with locally delivered autologous anti-FAP-targeted CAR T-cells. Ann. Oncol., 30, v501. 10.1093/annonc/mdz253.052

[B26] D'Arcangelo E. Wu N. C. Cadavid J. L. McGuigan A. P. (2020). The life cycle of cancer-associated fibroblasts within the tumour stroma and its importance in disease outcome. Br. J. cancer 122 (7), 931–942. 10.1038/s41416-019-0705-1 31992854 PMC7109057

[B27] De P. Aske J. Sulaiman R. Dey N. (2022). Bête noire of chemotherapy and targeted therapy: CAF-mediated resistance. Cancers 14, 1519. 10.3390/cancers14061519 35326670 PMC8946545

[B28] de Jonge M. J. Dumez H. Verweij J. Yarkoni S. Snyder D. Lacombe D. (2006). Phase I and pharmacokinetic study of halofuginone, an oral quinazolinone derivative in patients with advanced solid tumours. Eur. J. cancer 42 (12), 1768–1774. 10.1016/j.ejca.2005.12.027 16815702

[B168] de Visser K. E. Joyce J. A. (2023). The evolving tumor microenvironment: from cancer initiation to metastatic outgrowth. Cancer cell 41 (3), 374–403. 10.1016/j.ccell.2023.02.016 36917948

[B29] Deloch L. Fuchs J. Rückert M. Fietkau R. Frey B. Gaipl U. S. (2019). Low-dose irradiation differentially impacts macrophage phenotype in dependence of fibroblast-like synoviocytes and radiation dose. J. Immunol. Res. 2019, e3161750. 10.1155/2019/3161750 PMC6710796 31485459

[B171] Deng M. Lin J. Nowsheen S. Liu T. Zhao Y. Villalta P. W. (2020). Extracellular matrix stiffness determines DNA repair efficiency and cellular sensitivity to genotoxic agents. Science advances 6 (37), eabb2630. 10.1126/sciadv.abb2630 32917705 PMC7486107

[B30] Deng B. Zhao Z. Kong W. Han C. Shen X. Zhou C. (2022). Biological role of matrix stiffness in tumor growth and treatment. J. Transl. Med. 20, 540. 10.1186/s12967-022-03768-y 36419159 PMC9682678

[B31] Ding X. Xi W. Ji J. Cai Q. Jiang J. Shi M. (2018). HGF derived from cancer-associated fibroblasts promotes vascularization in gastric cancer via PI3K/AKT and ERK1/2 signaling. Oncol. Rep. 40, 1185–1195. 10.3892/or.2018.6500 29917165

[B32] Dong R. Guo J. Zhang Z. Zhou Y. Hua Y. (2018). Polyphyllin I inhibits gastric cancer cell proliferation by downregulating the expression of fibroblast activation protein alpha (FAP) and hepatocyte growth factor (HGF) in cancer-associated fibroblasts. Biochem. Biophysical Res. Commun. 497, 1129–1134. 10.1016/j.bbrc.2018.02.193 29499193

[B33] Elyada E. Bolisetty M. Laise P. Flynn W. F. Courtois E. T. Burkhart R. A. (2019). Cross-species single-cell analysis of pancreatic ductal adenocarcinoma reveals antigen-presenting cancer-associated fibroblasts. Cancer Discov. 9 (8), 1102–1123. 10.1158/2159-8290.CD-19-0094 31197017 PMC6727976

[B34] Emens L. A. Middleton G. (2015). The interplay of immunotherapy and chemotherapy: harnessing potential synergies. Cancer Immunol. Res. 3, 436–443. 10.1158/2326-6066.CIR-15-0064 25941355 PMC5012642

[B176] Fang J. -H. Zheng Z. -Y. Liu J. -Y. Xie C. Zhang Z. -J. Zhuang S. -M. (2017). Regulatory role of the MicroRNA-29b-IL-6 signaling in the formation of vascular mimicry. Mol. Ther. Nucleic Acids 8, 90–100. 10.1002/cac2.12392 28918059 PMC5493821

[B35] Fang Z. Meng Q. Xu J. Wang W. Zhang B. Liu J. (2022). Signaling pathways in cancer‐associated fibroblasts: recent advances and future perspectives. Cancer Commun. (Lond) 43, 3–41. 10.1002/cac2.12392 36424360 PMC9859735

[B36] Feng J. Tang Y. Xu Y. Sun Q. Liao F. Han D. (2013). Substrate stiffness influences the outcome of antitumor drug screening *in vitro* . Clin. Hemorheol. Microcirc. 55, 121–131. 10.3233/CH-131696 23445634

[B38] Finetti F. Travelli C. Ercoli J. Colombo G. Buoso E. Trabalzini L. (2020). Prostaglandin E2 and cancer: insight into tumor progression and immunity. Biology 9, 434. 10.3390/biology9120434 33271839 PMC7760298

[B39] Fozzatti L. Alamino V. A. Park S. Giusiano L. Volpini X. Zhao L. (2019). Interplay of fibroblasts with anaplastic tumor cells promotes follicular thyroid cancer progression. Sci. Rep. 9 (1), 8028. 10.1038/s41598-019-44361-6 31142771 PMC6541589

[B40] Fozzatti L. Cheng S. Y. (2020). Tumor cells and cancer-associated fibroblasts: a synergistic crosstalk to promote thyroid cancer. Endocrinol. Metab. Seoul. 35 (4), 673–680. 10.3803/EnM.2020.401 33161690 PMC7803596

[B41] Fuyuhiro Y. Yashiro M. Noda S. Kashiwagi S. Matsuoka J. Doi Y. (2011). Upregulation of cancer-associated myofibroblasts by TGF-β from scirrhous gastric carcinoma cells. Br. J. cancer 105 (7), 996–1001. 10.1038/bjc.2011.330 21863023 PMC3185946

[B42] Gao C. Liu F. Ye Q. Guo A. (2023). Cancer-associated fibroblasts affect tumor metabolism and immune microenvironment in gastric cancer and identification of its characteristic genes. J. Oncol. 2023, 1424589. 10.1155/2023/1424589 36755806 PMC9902124

[B43] Glabman R. A. Choyke P. L. Sato N. (2022). Cancer-associated fibroblasts: tumorigenicity and targeting for cancer therapy. Cancers 14, 3906. 10.3390/cancers14163906 36010899 PMC9405783

[B44] Glentis A. Oertle P. Mariani P. Chikina A. El Marjou F. Attieh Y. (2017). Cancer-associated fibroblasts induce metalloprotease-independent cancer cell invasion of the basement membrane. Nat. Commun. 8, 924. 10.1038/s41467-017-00985-8 29030636 PMC5640679

[B45] Gu L. Ding D. Wei C. Zhou D. (2023). Cancer-associated fibroblasts refine the classifications of gastric cancer with distinct prognosis and tumor microenvironment characteristics. Front. Oncol. 13, 1158863. 10.3389/fonc.2023.1158863 37404754 PMC10316023

[B46] Gunaydin G. (2021). CAFs interacting with TAMs in tumor microenvironment to enhance tumorigenesis and immune evasion. Front. Oncol. 11, 668349. 10.3389/fonc.2021.668349 34336660 PMC8317617

[B47] Ham I.-H. Oh H. J. Jin H. Bae C. A. Jeon S.-M. Choi K. S. (2019). Targeting interleukin-6 as a strategy to overcome stroma-induced resistance to chemotherapy in gastric cancer. Mol. Cancer 18, 68. 10.1186/s12943-019-0972-8 30927911 PMC6441211

[B48] Han M.-E. Kim H.-J. Shin D. H. Hwang S.-H. Kang C.-D. Oh S.-O. (2015). Overexpression of NRG1 promotes progression of gastric cancer by regulating the self-renewal of cancer stem cells. J. Gastroenterol. 50, 645–656. 10.1007/s00535-014-1008-1 25381017

[B49] Hasegawa T. Yashiro M. Nishii T. Matsuoka J. Fuyuhiro Y. Morisaki T. (2014). Cancer-associated fibroblasts might sustain the stemness of scirrhous gastric cancer cells via transforming growth factor-β signaling. Int. J. Cancer 134, 1785–1795. 10.1002/ijc.28520 24155219

[B50] He X.-J. Tao H.-Q. Hu Z.-M. Ma Y.-Y. Xu J. Wang H.-J. (2014). Expression of galectin-1 in carcinoma-associated fibroblasts promotes gastric cancer cell invasion through upregulation of integrin β1. Cancer Sci. 105, 1402–1410. 10.1111/cas.12539 25230369 PMC4462364

[B51] Hofheinz R. D. al-Batran S. E. Hartmann F. Hartung G. Jäger D. Renner C. (2003). Stromal antigen targeting by a humanised monoclonal antibody: an early phase II trial of sibrotuzumab in patients with metastatic colorectal cancer. Onkologie 26 (1), 44–48. 10.1159/000069863 12624517

[B52] Hong Z. Xie W. Zhuo H. Wei X. Wang K. Cheng J. (2023). Crosstalk between cancer cells and cancer-associated fibroblasts mediated by TGF-β1–IGFBP7 signaling promotes the progression of infiltrative gastric cancer. Cancers 15, 3965. 10.3390/cancers15153965 37568781 PMC10417438

[B53] Hu B. Wu C. Mao H. Gu H. Dong H. Yan J. (2022). Subpopulations of cancer-associated fibroblasts link the prognosis and metabolic features of pancreatic ductal adenocarcinoma. Ann. Transl. Med. 10, 262. 10.21037/atm-22-407 35402584 PMC8987890

[B54] Huang J. Tsang W.-Y. Li Z.-H. Guan X.-Y. (2023a). The origin, differentiation, and functions of cancer-associated fibroblasts in gastrointestinal cancer. Cell. Mol. Gastroenterology Hepatology 16, 503–511. 10.1016/j.jcmgh.2023.07.001 PMC10462789 37451403

[B55] Huang L. Wu R.-L. Xu A.-M. (2015). Epithelial-mesenchymal transition in gastric cancer. Am. J. Transl. Res. 7, 2141–2158. 26807164 PMC4697696

[B56] Huang Z. Byrd O. Tan S. Knight B. Lo G. Taylor L. (2023b). Periostin facilitates ovarian cancer recurrence by enhancing cancer stemness.30.534465. 10.1101/2023.03.30.534465 PMC10695946 38049490

[B57] Ishihara S. Haga H. (2022). Matrix stiffness contributes to cancer progression by regulating transcription factors. Cancers 14, 1049. 10.3390/cancers14041049 35205794 PMC8870363

[B58] Ishimoto T. Miyake K. Nandi T. Yashiro M. Onishi N. Huang K. (2017). Activation of transforming growth factor beta 1 signaling in gastric cancer-associated fibroblasts increases their motility, via expression of rhomboid 5 homolog 2, and ability to induce invasiveness of gastric cancer cells. Gastroenterology 153 (1), 191–204. 10.1053/j.gastro.2017.03.046 28390866

[B59] Italiano A. Verlingue L. Prenen H. Guerra E. M. Tosi D. Perets R. (2021). Clinical activity and safety of simlukafusp alfa, an engineered interleukin-2 variant targeted to fibroblast activation protein-α, combined with atezolizumab in patients with recurrent or metastatic cervical cancer. J. Clin. Oncol. 39 (15_Suppl. l), 5510. 10.1200/JCO.2021.39.15_suppl.5510

[B60] Izumi D. Ishimoto T. Miyake K. Sugihara H. Eto K. Sawayama H. (2016). CXCL12/CXCR4 activation by cancer-associated fibroblasts promotes integrin β1 clustering and invasiveness in gastric cancer. Int. J. Cancer 138, 1207–1219. 10.1002/ijc.29864 26414794

[B164] Janssen J. B. E. Medema J. P. Gootjes E. C. Tauriello D. V. F. Verheul H. M. W (2022). Mutant RAS and the tumor microenvironment as dual therapeutic targets for advanced colorectal cancer. Cancer treatment reviews 109, 102433. 10.1016/j.ctrv.2022.102433 35905558

[B61] Jayasingam S. D. Citartan M. Thang T. H. Mat Zin A. A. Ang K. C. Ch'ng E. S. (2020). Evaluating the polarization of tumor-associated macrophages into M1 and M2 phenotypes in human cancer tissue: technicalities and challenges in routine clinical practice. Front. Oncol. 9, 1512. 10.3389/fonc.2019.01512 32039007 PMC6992653

[B165] Jena B. C. Das C. K. Bharadwaj D. Mandal M. (2020). Cancer associated fibroblast mediated chemoresistance: a paradigm shift in understanding the mechanism of tumor progression. Biochimica et biophysica acta. Reviews on cancer 1874 (2), 188416. 10.1016/j.bbcan.2020.188416 32822826

[B62] Jeong H. Y. Ham I.-H. Lee S. H. Ryu D. Son S.-Y. Han S.-U. (2021). Spatially distinct reprogramming of the tumor microenvironment based on tumor invasion in diffuse-type gastric cancers. Clin. Cancer Res. 27, 6529–6542. 10.1158/1078-0432.CCR-21-0792 34385296

[B63] Jiang X. Liu F. Wang Y. Gao J. (2019). Secreted protein acidic and rich in cysteine promotes epithelial–mesenchymal transition of hepatocellular carcinoma cells and acquisition of cancerstem cell phenotypes. J. Gastroenterology Hepatology 34, 1860–1868. 10.1111/jgh.14692 31041810

[B166] Kasashima H. Yashiro M. Kinoshita H. Fukuoka T. Morisaki T. Masuda G. (2014). Lysyl oxidase-like 2 (LOXL2) from stromal fibroblasts stimulates the progression of gastric cancer. Cancer letters 354 (2), 438–446. 10.1016/j.canlet.2014.08.014 25128648

[B65] Kim J. Park C. Kim K. H. Kim E. H. Kim H. Woo J. K. (2022). Single-cell analysis of gastric pre-cancerous and cancer lesions reveals cell lineage diversity and intratumoral heterogeneity. npj Precis. Onc. 6, 9–11. 10.1038/s41698-022-00251-1 PMC8795238 35087207

[B66] Kim S.-K. Kim H.-J. Park J.-L. Heo H. Kim S.-Y. Lee S.-I. (2020). Identification of a molecular signature of prognostic subtypes in diffuse-type gastric cancer. Gastric Cancer 23, 473–482. 10.1007/s10120-019-01029-4 31773340 PMC7165151

[B67] Kitadai Y. (2010). Angiogenesis and lymphangiogenesis of gastric cancer. J. Oncol. 2010, e468725. 10.1155/2010/468725 PMC2847386 20369064

[B68] Kobayashi T. Chanmee T. Itano N. (2020). Hyaluronan: metabolism and function. Biomolecules 10, 1525. 10.3390/biom10111525 33171800 PMC7695009

[B69] Kogure A. Naito Y. Yamamoto Y. Yashiro M. Kiyono T. Yanagihara K. (2020). Cancer cells with high-metastatic potential promote a glycolytic shift in activated fibroblasts. PLoS One 15 (6), e0234613. 10.1371/journal.pone.0234613 32555715 PMC7299357

[B70] Kondělková K. Vokurková D. Krejsek J. Borská L. Fiala Z. Andrýs C. (2016). Regulatory T cells (Treg) and their roles in immune system with respect to immunopathological disorders. Acta Med. (Hradec Kralove, Czech Repub.) 53, 73–77. 10.14712/18059694.2016.63 20672742

[B167] Kondo R. Sakamoto N. Harada K. Hashimoto H. Morisue R. Yanagihara K. (2023). Cancer-associated fibroblast-dependent and -independent invasion of gastric cancer cells. J. Cancer Res. Clin. Oncol. 149, 5309–5319. 10.1007/s00432-022-04484-2 36416958 PMC11797592

[B71] Koppensteiner L. Mathieson L. O’Connor R. A. Akram A. R. (2022). Cancer associated fibroblasts - an impediment to effective anti-cancer T cell immunity. Front. Immunol. 13, 887380. 10.3389/fimmu.2022.887380 35479076 PMC9035846

[B72] Kudo A. Kii I. (2018). Periostin function in communication with extracellular matrices. J. Cell. Commun. Signal. 12, 301–308. 10.1007/s12079-017-0422-6 29086200 PMC5842185

[B73] Kwa M. Q. Herum K. M. Brakebusch C. (2019). Cancer-associated fibroblasts: how do they contribute to metastasis? Clin. Exp. Metastasis 36, 71–86. 10.1007/s10585-019-09959-0 30847799

[B74] Lauren P. (1965). THE TWO HISTOLOGICAL MAIN TYPES OF GASTRIC CARCINOMA: DIFFUSE AND SO-CALLED INTESTINAL-TYPE CARCINOMA. AN ATTEMPT AT A HISTO-CLINICAL CLASSIFICATION. Acta Pathol. Microbiol. Scand. 64, 31–49. 10.1111/apm.1965.64.1.31 14320675

[B75] Lavie D. Ben-Shmuel A. Erez N. Scherz-Shouval R. (2022). Cancer-associated fibroblasts in the single-cell era. Nat. Cancer 3, 793–807. 10.1038/s43018-022-00411-z 35883004 PMC7613625

[B78] Levental K. R. Yu H. Kass L. Lakins J. N. Egeblad M. Erler J. T. (2009). Matrix crosslinking forces tumor progression by enhancing integrin signaling. Cell. 139 (5), 891–906. 10.1016/j.cell.2009.10.027 19931152 PMC2788004

[B79] Li L. Wang X. (2021). Identification of gastric cancer subtypes based on pathway clustering. npj Precis. Onc. 5, 46–17. 10.1038/s41698-021-00186-z PMC8172826 34079012

[B80] Li L. Zhu Z. Zhao Y. Zhang Q. Wu X. Miao B. (2019). FN1, SPARC, and SERPINE1 are highly expressed and significantly related to a poor prognosis of gastric adenocarcinoma revealed by microarray and bioinformatics. Sci. Rep. 9, 7827. 10.1038/s41598-019-43924-x 31127138 PMC6534579

[B81] Li R. K. Zhao W. Y. Fang F. Zhuang C. Zhang X. X. Yang X. M. (2015). Lysyl oxidase-like 4 (LOXL4) promotes proliferation and metastasis of gastric cancer via FAK/Src pathway. J. cancer Res. Clin. Oncol. 141 (2), 269–281. 10.1007/s00432-014-1823-z 25216702 PMC11823919

[B82] Li X. Sun Z. Peng G. Xiao Y. Guo J. Wu B. (2022). Single-cell RNA sequencing reveals a pro-invasive cancer-associated fibroblast subgroup associated with poor clinical outcomes in patients with gastric cancer. Theranostics 12, 620–638. 10.7150/thno.60540 34976204 PMC8692898

[B83] Liberti M. V. Locasale J. W. (2016). The Warburg effect: how does it benefit cancer cells? Trends Biochem. Sci. 41 (3), 211–218. 10.1016/j.tibs.2015.12.001 26778478 PMC4783224

[B84] Lillie T. Parkes E. E. Ottensmeier C. Krige D. Ravanfar B. Evilevitch V. (2022). NEBULA: a multicenter phase 1a/b study of a tumor-selective transgene-expressing adenoviral vector, NG-641, and nivolumab in patients with metastatic or advanced epithelial tumors. J. Clin. Oncol. 40 (16_Suppl. l), TPS2682. 10.1200/JCO.2022.40.16_suppl.TPS2682

[B85] Lim J. R. Mouawad J. Gorton O. K. Bubb W. A. Kwan A. H. (2021). Cancer stem cell characteristics and their potential as therapeutic targets. Med. Oncol. 38, 76. 10.1007/s12032-021-01524-8 34050825

[B86] Lin X. Zhao Y. Song W. Zhang B. (2015). Molecular classification and prediction in gastric cancer. Comput. Struct. Biotechnol. J. 13, 448–458. 10.1016/j.csbj.2015.08.001 26380657 PMC4556804

[B87] Lin Y. Xu J. Lan H. (2019). Tumor-associated macrophages in tumor metastasis: biological roles and clinical therapeutic applications. J. Hematol. Oncol. 12, 76. 10.1186/s13045-019-0760-3 31300030 PMC6626377

[B88] Liu X. Yao L. Qu J. Liu L. Lu N. Wang J. (2021a). Cancer-associated fibroblast infiltration in gastric cancer: the discrepancy in subtypes pathways and immunosuppression. J. Transl. Med. 19 (1), 325. 10.1186/s12967-021-03012-z 34332586 PMC8325313

[B89] Liu Y. Zeng S. Hu Y. Zhang Y. Li J. (2021b). Overexpression of NREP promotes migration and invasion in gastric cancer through facilitating epithelial-mesenchymal transition. Front. Cell. Dev. Biol. 9, 746194. 10.3389/fcell.2021.746194 34746143 PMC8565479

[B173] Liu X. Li J. Yang X. Li X. Kong J. Qi D. (2023). Carcinoma-associated fibroblast-derived lysyl oxidase-rich extracellular vesicles mediate collagen crosslinking and promote epithelial-mesenchymal transition via p-FAK/p-paxillin/YAP signaling. Int J. Oral Sci. 15, 1–15. 10.1038/s41368-023-00236-1 37532712 PMC10397209

[B90] Loh J. J. Ma S. (2021). The role of cancer-associated fibroblast as a dynamic player in mediating cancer stemness in the tumor microenvironment. Front. Cell. Dev. Biol. 9, 727640. 10.3389/fcell.2021.727640 34760886 PMC8573407

[B91] Lou M. Iwatsuki M. Wu X. Zhang W. Matsumoto C. Baba H. (2023). Cancer-associated fibroblast-derived IL-8 upregulates PD-L1 expression in gastric cancer through the NF-κB pathway. Ann. Surg. Oncol. 10.1245/s10434-023-14586-x 38006530

[B92] Louault K. Li R.-R. DeClerck Y. A. (2020). Cancer-associated fibroblasts: understanding their heterogeneity. Cancers 12, 3108. 10.3390/cancers12113108 33114328 PMC7690906

[B93] Lu Y. Li D. Cao Y. Ying L. Tao Q. Xiong F. (2022). A genomic signature reflecting fibroblast infiltration into gastric cancer is associated with prognosis and treatment outcomes of immune checkpoint inhibitors. Front. Cell. Dev. Biol. 10, 862294. 10.3389/fcell.2022.862294 35557959 PMC9087633

[B94] Ma J. Song X. Xu X. Mou Y. (2018). Cancer-associated fibroblasts promote the chemo-resistance in gastric cancer through secreting IL-11 targeting JAK/STAT3/Bcl2 pathway. Cancer Res. Treat. 51, 194–210. 10.4143/crt.2018.031 29690750 PMC6333970

[B95] Ma Y. Zhu J. Chen S. Ma J. Zhang X. Huang S. (2019). Low expression of SPARC in gastric cancer-associated fibroblasts leads to stemness transformation and 5-fluorouracil resistance in gastric cancer. Cancer Cell. Int. 19, 137. 10.1186/s12935-019-0844-8 31139014 PMC6528188

[B96] Majidpoor J. Mortezaee K. (2021). Steps in metastasis: an updated review. Med. Oncol. 38, 3. 10.1007/s12032-020-01447-w 33394200

[B97] Mao X. Xu J. Wang W. Liang C. Hua J. Liu J. (2021). Crosstalk between cancer-associated fibroblasts and immune cells in the tumor microenvironment: new findings and future perspectives. Mol. Cancer 20, 131. 10.1186/s12943-021-01428-1 34635121 PMC8504100

[B98] Mezzapelle R. Leo M. Caprioglio F. Colley L. S. Lamarca A. Sabatino L. (2022). CXCR4/CXCL12 activities in the tumor microenvironment and implications for tumor immunotherapy. Cancers 14, 2314. 10.3390/cancers14092314 35565443 PMC9105267

[B99] Miki Y. Yashiro M. Moyano-Galceran L. Sugimoto A. Ohira M. Lehti K. (2020). Crosstalk between cancer associated fibroblasts and cancer cells in scirrhous type gastric cancer. Front. Oncol. 10, 568557. 10.3389/fonc.2020.568557 33178597 PMC7596590

[B100] Miyai Y. Esaki N. Takahashi M. Enomoto A. (2020). Cancer-associated fibroblasts that restrain cancer progression: hypotheses and perspectives. Cancer Sci. 111, 1047–1057. 10.1111/cas.14346 32060987 PMC7156845

[B101] Nallanthighal S. Heiserman J. P. Cheon D.-J. (2019). The role of the extracellular matrix in cancer stemness. Front. Cell. Dev. Biol. 7, 86. 10.3389/fcell.2019.00086 31334229 PMC6624409

[B102] Nie L. Lyros O. Medda R. Jovanovic N. Schmidt J. L. Otterson M. F. (2014). Endothelial-mesenchymal transition in normal human esophageal endothelial cells cocultured with esophageal adenocarcinoma cells: role of IL-1β and TGF-β2. Am. J. Physiology-Cell Physiology 307, C859–C877. 10.1152/ajpcell.00081.2014 PMC4216936 25163519

[B103] Ortiz-Otero N. Clinch A. B. Hope J. Wang W. Reinhart-King C. A. King M. R. (2020a). Cancer associated fibroblasts confer shear resistance to circulating tumor cells during prostate cancer metastatic progression. Oncotarget 11, 1037–1050. 10.18632/oncotarget.27510 32256977 PMC7105166

[B104] Ortiz-Otero N. Marshall J. R. Lash B. King M. R. (2020b). Chemotherapy-induced release of circulating-tumor cells into the bloodstream in collective migration units with cancer-associated fibroblasts in metastatic cancer patients. BMC Cancer 20, 873. 10.1186/s12885-020-07376-1 32917154 PMC7488506

[B105] Oshi M. Satyananda V. Angarita F. A. Kim T. H. Tokumaru Y. Yan L. (2021). Angiogenesis is associated with an attenuated tumor microenvironment, aggressive biology, and worse survival in gastric cancer patients. Am. J. Cancer Res. 11, 1659–1671. 33948380 PMC8085878

[B106] Park S.-G. Ji M.-J. Ham I.-H. Shin Y.-H. Lee S.-M. Lee C. H. (2023). Secretome analysis reveals reduced expression of COL4A2 in hypoxic cancer-associated fibroblasts with a tumor-promoting function in gastric cancer. J. Cancer Res. Clin. Oncol. 149, 4477–4487. 10.1007/s00432-022-04361-y 36125535 PMC11798037

[B107] Patten J. Wang K. (2021). Fibronectin in development and wound healing. Adv. Drug Deliv. Rev. 170, 353–368. 10.1016/j.addr.2020.09.005 32961203

[B172] Pei L. Liu Y. Liu L. Gao S. Gao X. Feng Y. (2023). Roles of cancer-associated fibroblasts (CAFs) in anti- PD-1/PD-L1 immunotherapy for solid cancers. Molecular cancer 22 (1), 29. 10.1186/s12943-023-01731-z 36759842 PMC9912573

[B109] Pines M. (2014). Halofuginone for fibrosis, regeneration and cancer in the gastrointestinal tract. World J. Gastroenterol. 20, 14778–14786. 10.3748/wjg.v20.i40.14778 25356039 PMC4209542

[B110] Ping Q. Yan R. Cheng X. Wang W. Zhong Y. Hou Z. (2021). Cancer-associated fibroblasts: overview, progress, challenges, and directions. Cancer Gene Ther. 28, 984–999. 10.1038/s41417-021-00318-4 33712707

[B111] Privé B. M. Boussihmad M. A. Timmermans B. van Gemert W. A. Peters S. M. B. Derks Y. H. W. (2023). Fibroblast activation protein-targeted radionuclide therapy: background, opportunities, and challenges of first (pre)clinical studies. Eur. J. Nucl. Med. Mol. Imaging 50, 1906–1918. 10.1007/s00259-023-06144-0 36813980 PMC10199876

[B112] Qin Y. Wang F. Ni H. Liu Y. Yin Y. Zhou X. (2021). Cancer-associated fibroblasts in gastric cancer affect malignant progression via the CXCL12-CXCR4 axis. J. Cancer 12, 3011–3023. 10.7150/jca.49707 33854601 PMC8040897

[B113] Quante M. Tu S. P. Tomita H. Gonda T. Wang S. S. W. Takashi S. (2011). Bone marrow-derived myofibroblasts contribute to the mesenchymal stem cell niche and promote tumor growth. Cancer Cell. 19, 257–272. 10.1016/j.ccr.2011.01.020 21316604 PMC3060401

[B114] Remsing Rix L. L. Sumi N. J. Hu Q. Desai B. Bryant A. T. Li X. (2022). IGF-binding proteins secreted by cancer-associated fibroblasts induce context-dependent drug sensitization of lung cancer cells. Sci. Signal. 15, eabj5879. 10.1126/scisignal.abj5879 35973030 PMC9528501

[B115] Scott A. M. Wiseman G. Welt S. Adjei A. Lee F. T. Hopkins W. (2003). A Phase I dose-escalation study of sibrotuzumab in patients with advanced or metastatic fibroblast activation protein-positive cancer. Clin. cancer Res. official J. Am. Assoc. Cancer Res. 9 (5), 1639–1647. 12738716

[B116] Sebastian A. Hum N. R. Martin K. A. Gilmore S. F. Peran I. Byers S. W. (2020). Single-cell transcriptomic analysis of tumor-derived fibroblasts and normal tissue-resident fibroblasts reveals fibroblast heterogeneity in breast cancer. Cancers 12, 1307. 10.3390/cancers12051307 32455670 PMC7281266

[B117] Setargew Y. F. I. Wyllie K. Grant R. D. Chitty J. L. Cox T. R. (2021). Targeting lysyl oxidase family meditated matrix cross-linking as an anti-stromal therapy in solid tumours. Cancers 13, 491. 10.3390/cancers13030491 33513979 PMC7865543

[B118] Sharma U. Medina-Saenz K. Miller P. C. Troness B. Spartz A. Sandoval-Leon A. (2021). Heterotypic clustering of circulating tumor cells and circulating cancer-associated fibroblasts facilitates breast cancer metastasis. Breast Cancer Res. Treat. 189, 63–80. 10.1007/s10549-021-06299-0 34216317

[B119] Singh P. Carraher C. Schwarzbauer J. E. (2010). Assembly of fibronectin extracellular matrix. Annu. Rev. Cell. Dev. Biol. 26, 397–419. 10.1146/annurev-cellbio-100109-104020 20690820 PMC3628685

[B120] Sükei T. Palma E. Urbani L. (2021). Interplay between cellular and non-cellular components of the tumour microenvironment in hepatocellular carcinoma. Cancers 13, 5586. 10.3390/cancers13215586 34771746 PMC8583132

[B121] Tang D. Gao J. Wang S. Ye N. Chong Y. Huang Y. (2016). Cancer-associated fibroblasts promote angiogenesis in gastric cancer through galectin-1 expression. Tumor Biol. 37, 1889–1899. –1899. 10.1007/s13277-015-3942-9 26323258

[B122] Teichgräber V. Monasterio C. Chaitanya K. Boger R. Gordon K. Dieterle T. (2015). Specific inhibition of fibroblast activation protein (FAP)-alpha prevents tumor progression *in vitro* . Adv. Med. Sci. 60, 264–272. 10.1016/j.advms.2015.04.006 26057860

[B123] Turrell F. K. Orha R. Guppy N. J. Gillespie A. Guelbert M. Starling C. (2023). Age-associated microenvironmental changes highlight the role of PDGF-C in ER+ breast cancer metastatic relapse. Nat. cancer 4 (4), 468–484. 10.1038/s43018-023-00525-y 36914817 PMC10132974

[B124] Ucaryilmaz Metin C. Ozcan G. (2022a). Comprehensive bioinformatic analysis reveals a cancer-associated fibroblast gene signature as a poor prognostic factor and potential therapeutic target in gastric cancer. BMC Cancer 22, 692. 10.1186/s12885-022-09736-5 35739492 PMC9229147

[B125] Ucaryilmaz Metin C. Ozcan G. (2022b). The HIF-1α as a potent inducer of the hallmarks in gastric cancer. Cancers 14, 2711. 10.3390/cancers14112711 35681691 PMC9179860

[B126] Ughetto S. Migliore C. Pietrantonio F. Apicella M. Petrelli A. D’Errico L. (2021). Personalized therapeutic strategies in HER2-driven gastric cancer. Gastric Cancer 24, 897–912. 10.1007/s10120-021-01165-w 33755862

[B169] van Meeteren L. A. ten Dijke P. (2012). Regulation of endothelial cell plasticity by TGF-β. Cell and Tissue Research 347 (1), 177–186. 10.1007/s00441-011-1222-6 21866313 PMC3250609

[B127] Vu L. T. Peng B. Zhang D. X. Ma V. Mathey-Andrews C. A. Lam C. K. (2019). Tumor-secreted extracellular vesicles promote the activation of cancer-associated fibroblasts via the transfer of microRNA-125b. J. Extracell. Vesicles 8 (1), 1599680. 10.1080/20013078.2019.1599680 31044053 PMC6484490

[B128] Walcher L. Kistenmacher A.-K. Suo H. Kitte R. Dluczek S. Strauß A. (2020). Cancer stem cells—origins and biomarkers: perspectives for targeted personalized therapies. Front. Immunol. 11, 1280. 10.3389/fimmu.2020.01280 32849491 PMC7426526

[B129] Waldhauer I. Gonzalez-Nicolini V. Freimoser-Grundschober A. Nayak T. K. Fahrni L. Hosse R. J. (2021). Simlukafusp alfa (FAP-IL2v) immunocytokine is a versatile combination partner for cancer immunotherapy. mAbs 13 (1), 1913791. 10.1080/19420862.2021.1913791 33974508 PMC8115765

[B130] Wang B. Xu Y.-F. He B.-S. Pan Y.-Q. Zhang L.-R. Zhu C. (2010). RNAi-mediated silencing of CD147 inhibits tumor cell proliferation, invasion and increases chemosensitivity to cisplatin in SGC7901 cells *in vitro* . J. Exp. Clin. Cancer Res. 29, 61. 10.1186/1756-9966-29-61 20525232 PMC2893454

[B131] Wang Y. Gong T. Zhang Z.-R. Fu Y. (2017). Matrix stiffness differentially regulates cellular uptake behavior of nanoparticles in two breast cancer cell lines. ACS Appl. Mat. Interfaces 9, 25915–25928. 10.1021/acsami.7b08751 28718278

[B174] Wang Y. Xu X. Marshall J. E. Gong M. Zhao Y. Dua K. (2021a). Loss of hyaluronan and proteoglycan link protein-1 induces tumorigenesis in colorectal cancer. Front. Oncology 11. Available at: https://www.frontiersin.org/journals/oncology/articles/10.3389/fonc.2021.754240 (Accessed January 21, 2024). 10.3389/fonc.2021.754240 PMC8710468 34966673

[B132] Wang Z. Yang Q. Tan Y. Tang Y. Ye J. Yuan B. (2021). Cancer-associated fibroblasts suppress cancer development: the other side of the coin. Front. Cell. Dev. Biol. 9, 613534. 10.3389/fcell.2021.613534 33614646 PMC7890026

[B133] Wei J. Yao J. Yan M. Xie Y. Liu P. Mao Y. (2022). The role of matrix stiffness in cancer stromal cell fate and targeting therapeutic strategies. Acta Biomater. 150, 34–47. 10.1016/j.actbio.2022.08.005 35948177

[B134] Wei R. Song J. Liu X. Huo S. Liu C. Liu X. (2023). Immunosuppressive MFAP2+ cancer associated fibroblasts conferred unfavorable prognosis and therapeutic resistance in gastric cancer. Cell. Oncol. 10.1007/s13402-023-00849-y PMC12973947 37540308

[B135] Wells R. G. (2013). Tissue mechanics and fibrosis. Biochimica Biophysica Acta (BBA)-Mol. Basis Dis. 1832, 884–890. 10.1016/j.bbadis.2013.02.007 PMC3641165 23434892

[B136] Winkler J. Abisoye-Ogunniyan A. Metcalf K. J. Werb Z. (2020). Concepts of extracellular matrix remodelling in tumour progression and metastasis. Nat. Commun. 11, 5120. 10.1038/s41467-020-18794-x 33037194 PMC7547708

[B175] Wu C. Li D. Cheng X. Gu H. Qian Y. Feng L. (2023). Downregulation of cancer-associated fibroblast exosome-derived miR-29b-1-5p restrains vasculogenic mimicry and apoptosis while accelerating migration and invasion of gastric cancer cells via immunoglobulin domain-containing 1/zonula occluden-1 axis. Cell Cycle 22, 1807–1826. 10.1080/15384101.2023.2231740 37587724 PMC10599179

[B137] Wu F. Yang J. Liu J. Wang Y. Mu J. Zeng Q. (2021). Signaling pathways in cancer-associated fibroblasts and targeted therapy for cancer. Sig Transduct. Target Ther. 6, 218–235. 10.1038/s41392-021-00641-0 PMC8190181 34108441

[B138] Wu X. Tao P. Zhou Q. Li J. Yu Z. Wang X. (2017). IL-6 secreted by cancer-associated fibroblasts promotes epithelial-mesenchymal transition and metastasis of gastric cancer via JAK2/STAT3 signaling pathway. Oncotarget 8, 20741–20750. 10.18632/oncotarget.15119 28186964 PMC5400541

[B139] Wynn T. A. Ramalingam T. R. (2012). Mechanisms of fibrosis: therapeutic translation for fibrotic disease. Nat. Med. 18, 1028–1040. 10.1038/nm.2807 22772564 PMC3405917

[B140] Xie Q. Ding J. Chen Y. (2021). Role of CD8+ T lymphocyte cells: interplay with stromal cells in tumor microenvironment. Acta Pharm. Sin. B 11, 1365–1378. 10.1016/j.apsb.2021.03.027 34221857 PMC8245853

[B141] Xing F. Saidou J. Watabe K. (2010). Cancer associated fibroblasts (CAFs) in tumor microenvironment. FBL 15, 166–179. 10.2741/3613 20036813 PMC2905156

[B142] Yamaguchi H. Yoshida N. Takanashi M. Ito Y. Fukami K. Yanagihara K. (2014). Stromal fibroblasts mediate extracellular matrix remodeling and invasion of scirrhous gastric carcinoma cells. PLOS ONE 9, e85485. 10.1371/journal.pone.0085485 24427313 PMC3888433

[B143] Yang D. Liu J. Qian H. Zhuang Q. (2023). Cancer-associated fibroblasts: from basic science to anticancer therapy. Exp. Mol. Med. 55 (7), 1322–1332. 10.1038/s12276-023-01013-0 37394578 PMC10394065

[B144] Yang S. S. Ma S. Dou H. Liu F. Zhang S. Y. Jiang C. (2020). Breast cancer-derived exosomes regulate cell invasion and metastasis in breast cancer via miR-146a to activate cancer associated fibroblasts in tumor microenvironment. Exp. Cell. Res. 391 (2), 111983. 10.1016/j.yexcr.2020.111983 32268136

[B170] Yang Y. Ye W. L. Zhang R. N. He X. S. Wang J. R. Liu Y. X. (2021). The role of TGF-β signaling pathways in cancer and its potential as a therapeutic target. Evid Based Complement Alternat Med 2021, 6675208. 10.1155/2021/6675208 34335834 PMC8321733

[B145] Yang Y. Ma Y. Yan S. Wang P. Hu J. Chen S. (2022). CAF promotes chemoresistance through NRP2 in gastric cancer. Gastric Cancer 25, 503–514. 10.1007/s10120-021-01270-w 34826008 PMC9013334

[B147] Yu B. Chen X. Li J. Qu Y. Su L. Peng Y. (2013). Stromal fibroblasts in the microenvironment of gastric carcinomas promote tumor metastasis via upregulating TAGLN expression. BMC Cell. Biol. 14, 17. 10.1186/1471-2121-14-17 23510049 PMC3610155

[B149] Yu Y. Xiao C.-H. Tan L.-D. Wang Q.-S. Li X.-Q. Feng Y.-M. (2014). Cancer-associated fibroblasts induce epithelial-mesenchymal transition of breast cancer cells through paracrine TGF-β signalling. Br. J. Cancer 110, 724–732. 10.1038/bjc.2013.768 24335925 PMC3915130

[B150] Yusuf N. Inagaki T. Kusunoki S. Okabe H. Yamada I. Matsumoto A. (2014). SPARC was overexpressed in human endometrial cancer stem-like cells and promoted migration activity. Gynecol. Oncol. 134, 356–363. 10.1016/j.ygyno.2014.04.009 24769035

[B151] Zeng D. Li M. Zhou R. Zhang J. Sun H. Shi M. (2019). Tumor microenvironment characterization in gastric cancer identifies prognostic and immunotherapeutically relevant gene signatures. Cancer Immunol. Res. 7, 737–750. 10.1158/2326-6066.CIR-18-0436 30842092

[B152] Zhai J. Shen J. Xie G. Wu J. He M. Gao L. (2019). Cancer-associated fibroblasts-derived IL-8 mediates resistance to cisplatin in human gastric cancer. Cancer Lett. 454, 37–43. 10.1016/j.canlet.2019.04.002 30978440

[B153] Zhang C. Fei Y. Wang H. Hu S. Liu C. Hu R. (2023a). CAFs orchestrates tumor immune microenvironment—a new target in cancer therapy? Front. Pharmacol. 14, 1113378. 10.3389/fphar.2023.1113378 37007004 PMC10064291

[B154] Zhang D. Sun R. Di C. Li L. Zhao F. Han Y. (2023b). Microdissection of cancer-associated fibroblast infiltration subtypes unveils the secreted SERPINE2 contributing to immunosuppressive microenvironment and immuotherapeutic resistance in gastric cancer: a large-scale study integrating bulk and single-cell transcriptome profiling. Comput. Biol. Med. 166, 107406. 10.1016/j.compbiomed.2023.107406 37729702

[B155] Zhang H. Ren L. Ding Y. Li F. Chen X. Ouyang Y. (2019). Hyaluronan-mediated motility receptor confers resistance to chemotherapy via TGFβ/Smad2-induced epithelial-mesenchymal transition in gastric cancer. FASEB J. 33, 6365–6377. 10.1096/fj.201802186R 30802150

[B156] Zhang T. Li X. He Y. Wang Y. Shen J. Wang S. (2022). Cancer-associated fibroblasts-derived HAPLN1 promotes tumour invasion through extracellular matrix remodeling in gastric cancer. Gastric Cancer 25, 346–359. 10.1007/s10120-021-01259-5 34724589 PMC8882084

[B157] Zhao Z. Zhang Y. Guo E. Zhang Y. Wang Y. (2023a). Periostin secreted from podoplanin-positive cancer-associated fibroblasts promotes metastasis of gastric cancer by regulating cancer stem cells via AKT and YAP signaling pathway. Mol. Carcinog. 62, 685–699. 10.1002/mc.23517 36785937

[B158] Zhao Z. Zhang Y. Sun H. Chen Z. Chang J. Wang X. (2023b). Calcipotriol abrogates cancer-associated fibroblast-derived IL-8-mediated oxaliplatin resistance in gastric cancer cells via blocking PI3K/Akt signaling. Acta Pharmacol. Sin. 44, 178–188. 10.1038/s41401-022-00927-1 35676532 PMC9813133

[B159] Zheng H. Liu H. Ge Y. Wang X. (2021). Integrated single-cell and bulk RNA sequencing analysis identifies a cancer associated fibroblast-related signature for predicting prognosis and therapeutic responses in colorectal cancer. Cancer Cell. Int. 21, 552. 10.1186/s12935-021-02252-9 34670584 PMC8529760

[B160] Zhi K. Shen X. Zhang H. Bi J. (2010). Cancer-associated fibroblasts are positively correlated with metastatic potential of human gastric cancers. J. Exp. Clin. Cancer Res. 29, 66. 10.1186/1756-9966-29-66 20529313 PMC2892440

[B161] Zhou Z. Wei J. Kuang L. Zhang K. Liu Y. He Z. (2022). Characterization of aging cancer-associated fibroblasts draws implications in prognosis and immunotherapy response in low-grade gliomas. Front. Genet. 13, 897083. 10.3389/fgene.2022.897083 36092895 PMC9449154

[B162] Zhu P. Lu H. Wang M. Chen K. Chen Z. Yang L. (2023). Targeted mechanical forces enhance the effects of tumor immunotherapy by regulating immune cells in the tumor microenvironment. Cancer Biol. Med. 20, 44–55. 10.20892/j.issn.2095-3941.2022.0491 36647779 PMC9843446

[B163] Zhu Q. Zhang X. Zhang L. Li W. Wu H. Yuan X. (2014). The IL-6-STAT3 axis mediates a reciprocal crosstalk between cancer-derived mesenchymal stem cells and neutrophils to synergistically prompt gastric cancer progression. Cell. death Dis. 5 (6), e1295. 10.1038/cddis.2014.263 24946088 PMC4611735

